# Parental epigenetic age acceleration and risk of adverse birth outcomes: the Norwegian mother, father and child cohort study

**DOI:** 10.1186/s12916-024-03780-7

**Published:** 2024-11-25

**Authors:** Maria C. Magnus, Yunsung Lee, Ellen Ø. Carlsen, Lise A. Arge, Astanand Jugessur, Liv G. Kvalvik, Nils-Halvdan Morken, Cecilia H. Ramlau-Hansen, Mikko Myrskylä, Per Magnus, Siri E. Håberg

**Affiliations:** 1 Centre for Fertility and Health, Norwegian Institute of Public Health, P.O. Box 222 Skøyen, Oslo, 0213 Norway; 2 Department of Global Public Health and Primary Care, University of Bergen, Bergen, Norway; 3 Department of Obstetrics and Gynecology, Haukeland University Hospital, Bergen, Norway; 4 Department of Public Health, Research Unit for Epidemiology, Aarhus University, Aarhus, Denmark; 5 Max Planck Institute for Demographic Research, Rostock, Germany; 6 Helsinki Institute for Demography and Population Health, University of Helsinki, Helsinki, Finland; 7 Max Planck, University of Helsinki Center for Social Inequalities in Population Health, Rostock, Germany

**Keywords:** Epigenetic age, Adverse birth outcomes, Gestational age, Birthweight, Pre-eclampsia

## Abstract

**Background:**

Few studies have examined associations between maternal epigenetic age acceleration and adverse birth outcomes, and none have investigated paternal epigenetic age acceleration. Our objective was to assess the associations of parental (both maternal and paternal) epigenetic age acceleration in relation to birth outcomes.

**Methods:**

Parental epigenetic age was estimated using seven established epigenetic clocks in 2198 mothers and 2193 fathers from the Norwegian Mother, Father, and Child Cohort Study (MoBa). Individual epigenetic age acceleration was then calculated as residuals from linear regressions of estimates from the epigenetic clocks on chronological age. Further, linear regression was used to analyze differences in continuous outcomes (gestational length and standardized birthweight), while logistic regression was used for binary outcomes (preterm birth, post-term birth, small-for-gestational age [SGA], large-for-gestational age [LGA], and pre-eclampsia), adjusting for chronological age, parity, educational level, smoking, and BMI.

**Results:**

Increasing maternal, but not paternal, epigenetic age acceleration was associated with decreased gestational length for five out of six clocks, with adjusted estimates ranging from a mean 0.51-day decrease (95% CI − 1.00, − 0.02; *p*-value 0.043) for the Horvath clock to a 0.80-day decrease (95% CI − 1.29, − 0.31; *p*-value 0.002) for the Levine clock. An association with increasing maternal epigenetic age acceleration according to the DunedinPACE clock was also seen with greater standardized birthweight [mean difference 0.08 (95% CI 0.04, 0.12; *p*-value < 0.001]. These results were also reflected in an increased risk of spontaneous preterm birth and LGA. No associations were observed with post-term birth, SGA, or pre-eclampsia.

**Conclusions:**

Maternal, but not paternal, epigenetic age acceleration is associated with shorter pregnancies and an increased risk of spontaneous preterm birth. This may suggest that women’s biological age acceleration, including factors such as metabolic and physiologic state, is an additional risk factor for preterm delivery, beyond chronological age.

**Supplementary Information:**

The online version contains supplementary material available at 10.1186/s12916-024-03780-7.

## Background

The risk of most adverse birth outcomes, including stillbirth, preterm birth, and poor intrauterine growth, increases with maternal age [1, 2]. A similar, though less pronounced, increase in risk has been observed with increasing paternal age [3, 4], although it is difficult to fully disentangle the relative contributions, as partners are typically relatively close in age. These risks may reflect the impact of aging on placentation, vascular dysfunction, genetic quality of the oocyte/sperm in aging parents, among other factors [1–4]. Notably, individuals age at different rates biologically. Different indices of biological aging have been proposed [5], among which epigenetic aging estimated by DNA methylation patterns is one of the most commonly used to gauge biological aging [6]. Less research has been conducted on whether biological aging rather than chronological aging might have a greater impact on the risk of adverse birth outcomes.

Several epigenetic aging clocks have been developed in recent years. The first generation of these clocks, based on DNA-methylation measurements at cytosine-phosphate-guanine dinucleotide motifs (CpG sites) across the genome, were found to precisely predict chronological age [7, 8]. Individuals who are epigenetically older than their chronological age appear to have an increased risk of various chronic diseases and a shorter life expectancy [9]. The second generation of epigenetic aging clocks were developed to predict age-related diseases, life expectancy, and the pace of biological aging with greater precision than the first-generation clocks [10–12]. In the context of birth outcome risks, epigenetic clocks may potentially reflect the parents’ metabolic and physiological state [7, 8, 10–12]. Few studies have evaluated differences in birth outcomes according to maternal estimates of epigenetic age [13–18]. These studies are typically small, show conflicting findings, do not adequately adjust for background characteristics, and have only evaluated maternal and not paternal epigenetic age. The most commonly evaluated pregnancy outcome is gestational length [13, 15, 17, 18], while some studies also examined birthweight [17, 18] and pre-eclampsia [14, 16]. We found only one study that investigated the relationship between paternal epigenetic age estimated from sperm and the risk of birth outcomes [19].

To mitigate this knowledge gap, our objective was to examine associations of parental (both maternal and paternal) epigenetic age acceleration in relation to birth outcome.

## Methods

### Study population

**Fig. 1:**
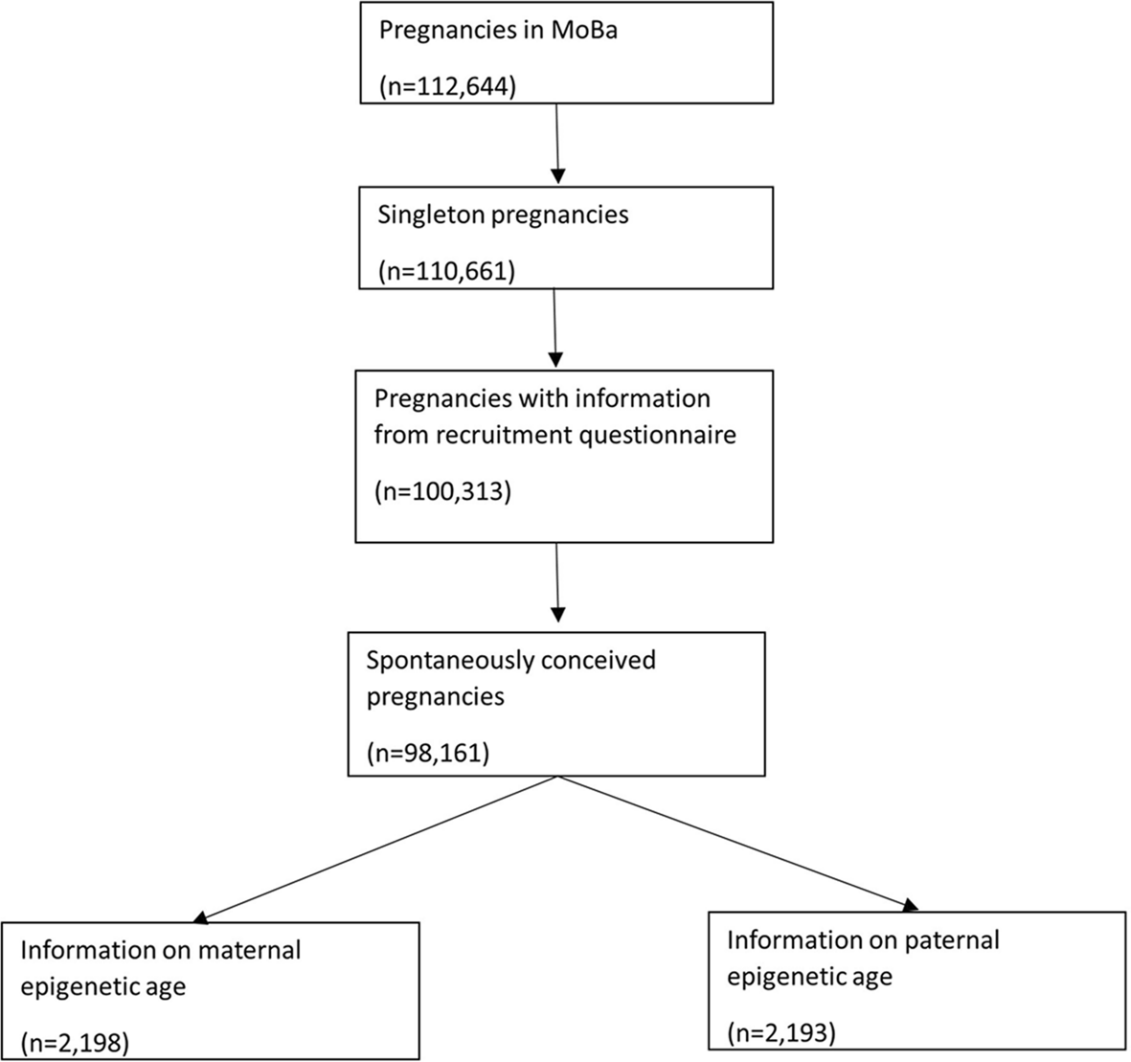
Illustration of study population

The Norwegian Mother, Father and Child Cohort Study (MoBa) is a population-based pregnancy cohort study [20]. Participants were recruited from all over Norway from 1999 to 2008. The women consented to participation in 41% of the pregnancies, and fathers were invited from 2001 onwards. The cohort includes approximately 114,500 children, 95,200 mothers, and 75,200 fathers. Using a unique personal identification number assigned to residents in Norway, data from questionnaires were linked to information from the Medical Birth Registry of Norway (MBRN). Blood was drawn from both parents at recruitment, at around gestational week 18 [21]. This study included a subset of 2198 randomly selected spontaneously conceived singleton pregnancies with information on maternal epigenetic age, out of which 2193 also had information on paternal epigenetic age. The selection of eligible couples is shown in Fig. 1.

### Measures of epigenetic age acceleration

The measurement of peripheral blood-derived DNA-methylation in the current dataset is described in detail elsewhere [22]. Briefly, methylation was measured using the Illumina Infinium MethylationEPIC V1 Array (Illumina, San Diego, CA, USA) [23]. After quality control, 770,586 autosomal probes remained and were used to calculate epigenetic aging. Epigenetic age was estimated by averaging DNA-methylation levels across a range of cell types at selected CpGs. Regarding the seven epigenetic age clocks used in this study: the Hannum, Horvath (pan-tissue), Levine and PCGrimAge clocks, estimate epigenetic age (in years) at sampling time [7, 8, 11, 12, 24], while the DNAmTL clock estimates telomere length (in kb) at sampling time [25], and the DunedinPACE clocks reflect the pace of aging (in years) over the past 10–15 years at sampling time [26]. Despite these variations, the epigenetic age estimates were treated in the same way in our analyses. The epigenetic biomarkers of aging were derived manually by taking weighted averages over the DNA methylation levels at selected CpGs. The weights, i.e., the coefficient estimates from penalized regressions, were sourced from the previous publications of the epigenetic age clocks [7, 8, 10, 11, 25]. Some CpGs were excluded because they did not pass the quality control procedures described previously [22]. The number of excluded CpGs was as follows: 6 out of 513 for the Levine clock [11], 21 out of 140 for DNAmTL clock [25], 24 out of 353 for the Horvath clock [8], and 9 out of 71 for the Hannum clock [7]. PCGrimAge [24] was calculated using the R code available on Higgins-Chen et al.’s GitHub repository: https://github.com/MorganLevineLab/PC-Clocks. For DunedinPACE [26], we used Belsky et al.’s GitHub repository: https://github.com/danbelsky/DunedinPACE. To obtain a measure of epigenetic age acceleration, we performed a linear regression of each epigenetic age estimate against chronological age at blood sampling. The resulting residual terms were standardized to *Z*-scores, so that negative *Z*-scores denote epigenetic age deceleration, and positive *Z*-scores denote epigenetic age acceleration. We show all estimates as the differences in the outcome per standard deviation increase in epigenetic age acceleration.

### Adverse birth outcomes

Information on birth outcomes was retrieved from the MBRN. This included information on gestational length in days (estimated by ultrasound [95% of pregnancies] or last menstrual period), birthweight in grams, and preeclampsia (including registrations of preeclampsia, eclampsia and “Hemolysis, Elevated Liver enzymes, and Low Platelet” [HELLP] syndrome). Birthweight was standardized to *Z*-scores by sex and gestational length. We defined preterm birth as birth before 37 completed gestational weeks, post term birth as birth after 42 completed gestational weeks, small-for-gestational-age (SGA) as birthweight less than the 10th percentile by sex and gestational length, and large-for-gestational-age (LGA) as birthweight greater than the 90th percentile by sex and gestational length. The percentiles used to estimate small and large for gestational length were based on the entire MoBa cohort. We further examined spontaneous preterm birth specifically, defined as onset of labor not registered as initiated by caesarean section or other interventions.

### Covariates

We obtained additional information on parental background characteristics collected from MoBa questionnaires at the time of recruitment/same time as blood draws. These included body mass index (BMI, pre-pregnancy for women and current for men, categorized as < 25/25–29.9/ ≥ 30 kg/m^2^), cigarette smoking status (no/former/current), highest completed or ongoing educational level (less than high school/high school/up to 4 years of college/more than four years of college), chronic hypertension (yes vs. no), diabetes mellitus (yes vs. no), and gestational diabetes (yes vs. no) for mothers. We included missing categories for education, smoking, and BMI. We did not include an underweight category for BMI due to small numbers. For smoking status in women, we had information on self-reported smoking status during the 3 months prior to pregnancy. For smoking status in men, we had information on self-reported smoking status during 6 months prior to pregnancy. This is due to differences in the way the retrospective questions regarding smoking were posed to women and men.

### Statistical analyses

For continuous outcomes, we used linear regression to examine the mean difference in gestational length and standardized birthweight with respect to increasing epigenetic age acceleration. For binary outcomes, we used logistic regression. We examined associations with one standard deviation increase in maternal and paternal epigenetic age acceleration derived from the seven epigenetic age clocks separately. The main analysis adjusted for chronological age only. In sensitivity analyses, we adjusted for cell type proportions and gestational week of blood sampling, in addition to maternal parity, and parental (maternal or paternal) education, smoking, BMI, diabetes, and chronic hypertension. Cell type proportions were estimated from blood-derived DNAm data using the minfi R package and the FlowSorted.Blood.EPIC reference dataset [27]. We used the estimateCellCounts2 function with preprocessNoob for background correction and an iterative algorithm for Identifying Optimal Libraries (IDOL) for probe selection.

A sensitivity analysis was conducted using the gestational length estimated by the last menstrual period instead of ultrasound measurements. Furthermore, we conducted a sensitivity analysis of the risk of post-term delivery using Cox analysis, starting the follow-up when the pregnancies reached term (day 37 + 0), and censoring births that were induced or initiated by caesarean section. We also examined an interaction with chronological age, by conducting a stratified analysis according to whether their chronological age was below or above 30 years of age, in addition to formally testing this by adding a product term in the regression model. To evaluate potential non-linear relationships, we conducted an analysis categorizing the standardized measures of epigenetic age acceleration into deceleration (< −0.5), acceleration (> 0.5), and neither (between − 0.5 and 0.5; reference). We then tested for a non-linear relationship by including a second-order term for the measures of epigenetic age acceleration in the model. We also conducted exploratory analyses stratifying by offspring sex due to known sex difference in birth outcomes.

All analyses were conducted using Stata version 17 (Statacorp, Texas).

## Results

The distribution of parental background characteristics among eligible singleton spontaneously conceived pregnancies and those with measurements of maternal and paternal epigenetic age were similar (Additional file 1: Table S1). For pregnancies with information on maternal epigenetic age acceleration, the mean age was 30 years (SD 5), 27% had more than 4 years of higher education, 29% smoked during the last 3 months prior to pregnancy, and 33% were overweight/obese (Table 1). For pregnancies with information on paternal epigenetic age, mean paternal age was 33 years (SD 5), 23% had four or more years of higher education, 29% smoked in the past 6 months before pregnancy, and 54% were overweight/obese (Table 1). The relationship between the epigenetic age estimates and chronological age is shown in Additional file 1: Table S2. The correlation between the epigenetic age estimates from the different clocks ranged from 0.02 to 0.61 (Additional file 1: Tables S3 and S4). The correlations between partners’ epigenetic age estimates ranged from 0.12 to 0.67 (Additional file 1: Table S5).

**Table 1:** Background characteristics of mothers and father with information on epigenetic age

Background characteristics	Pregnancies with maternal epigenetic age information (*n* = 2198)	Pregnancies with paternal epigenetic age information (*n* = 2193)
**Age at measurement of epigenetic age, mean (SD)**	30.1 (4.5)	32.7 (5.4)
**Gestational week of measurement, median (IQR)**	19 (18, 20)	19 (18, 20)
**Educational level, *n*(%)**		
Less than high-school	106 (4.8)	164 (7.5)
High-school	588 (26.8)	830 (37.9)
College, up to 4 years	914 (41.6)	623 (28.4)
College, more than 4 years	584 (26.6)	512 (23.4)
Missing	6 (0.3)	64 (2.9)
**Primiparous, *n*(%)**		
No	1170 (53.2)	NA
Yes	1028 (46.8)	NA
**Smoking status, *n*(%)**^a^		
Never	1118 (50.9)	1016 (46.3)
Former	442 (20.1)	404 (18.4)
Current	628 (28.6)	637 (29.1)
Missing	10 (0.5)	136 (6.2)
**Body mass index, *n*(%)**		
< 25	1454 (66.6)	906 (41.3)
25–29.9	474 (21.6)	966 (44.1)
≥ 30	211 (9.6)	226 (10.3)
Missing	49 (2.2)	95 (4.3)
**Diabetes mellitus, *n*(%)**		
No	2179 (99.1)	2172 (99.1)
Yes	19 (0.9)	20 (0.9)
**Chronic hypertension, *n*(%)**		
No	2176 (99.0)	2130 (97.1)
Yes	22 (1.0)	63 (2.9)
**Gestational diabetes, *n*(%)**		
No	2171 (98.2)	NA
Yes	27 (1.2)	NA

^a^Reflects the last 3 months prior to pregnancy for mothers and the last 6 months prior to pregnancy for fathers

**Fig. 2:**
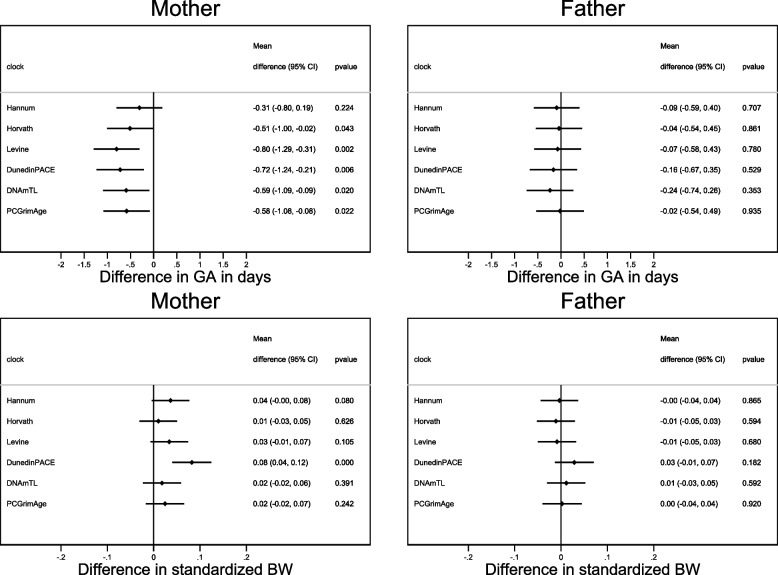
Adjusted mean difference in gestational length (GA) and standardized birthweight (BW) according to parental epigenetic age acceleration. Adjusted for parental age, parity, education, smoking and body-mass index

The mean gestational length was 280 days (SD 12) and the mean birthweight was 3638 g (SD 538) in pregnancies with information on maternal epigenetic age acceleration. Local linear smoothed plots of gestational length and standardized birthweight according to estimates of maternal and paternal epigenetic age acceleration are shown in Additional file 1: Figures S1-S4; there was no clear evidence of non-linear relationships (*p*-values for second order terms > 0.1). Increasing maternal epigenetic age acceleration showed a consistent association with decreasing mean gestational length for five out of the six clocks evaluated, with adjusted estimates ranging from a mean 0.51-day decrease (95% CI − 1.00, − 0.02) for the Horvath clock to a 0.80-day decrease (95% CI − 1.29, − 0.31) for the Levine clock (Fig. 2). Maternal epigenetic age acceleration according to the DunedinPACE clock was also associated with greater standardized birthweight, with a mean increase of 0.08 (95% CI 0.04, 0.12), although a similar tendency was not observed for any of the other clocks (Fig. 2). Similar associations between paternal epigenetic age acceleration and decreased gestational length were not observed (Fig. 2).

**Fig. 3:**
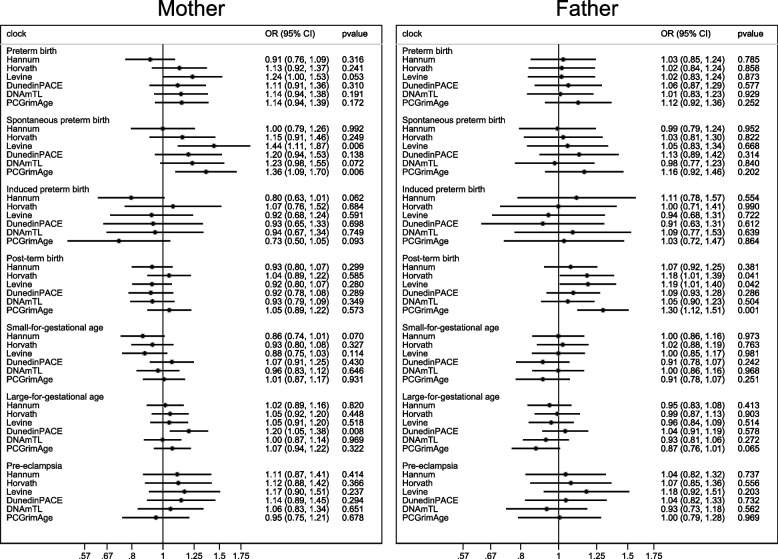
Risk of adverse birth outcomes according to parental epigenetic age acceleration. Adjusted for parental age, parity, education, smoking and body-mass index

Five percent of pregnancies were preterm, 3% spontaneously preterm, 8% post-term, and 3% of pregnancies were exposed to preeclampsia among pregnancies with information on maternal epigenetic age acceleration (Additional file 1: Table S6). In line with the reduction in gestational length with increasing maternal epigenetic age acceleration, we observed a tendency for an increased risk of preterm birth across most clocks, although the confidence intervals were wide (Fig. 3). A more prominent increased risk with increasing maternal epigenetic age acceleration was seen for spontaneous preterm birth, with an OR of 1.44 (96% CI 1.11, 1.87) per standard deviation increase in the estimate for the Levine clock, and OR of 1.36 (95% CI 1.09, 1.70) for the PCGrimAge clock (Fig. 3). Maternal epigenetic age acceleration according to the DunedinPACE clock was further associated with an increased risk of large-for-gestational age (OR 1.20; 95% CI 1.05, 1.38) (Fig. 3). An increased risk of post-term birth was seen with increasing paternal epigenetic age acceleration based on the Hannum (OR 1.18; 95% CI 1.01, 1.39), Levine (OR 1.19; 95% CI 1.01, 1.40), and PCGrimAge (OR 1.30; 95% CI 1.12, 1.51) clocks (Fig. 3). No notable associations were observed between maternal or paternal epigenetic age acceleration with the risk of SGA or the risk of pre-eclampsia (Fig. 3).

If we apply a Bonferroni correction for multiple testing, accounting for the evaluation of six epigenetic age clocks in women and men (*p*-value 0.05/12 = 0.004), only the association between maternal epigenetic age acceleration according to the Levine clock and reduced gestational age remained statistically significant (*p*-value 0.002). Additional adjustment for cell type composition and gestational week of blood sampling only slightly attenuated the results (Additional file 1: Figure S5 and S6), while adjustment for parity, education, smoking, BMI, diabetes, and chronic hypertension did not notably change our findings (Additional file 1: Figures S7 and S8). Mutual adjustment for the partner’s epigenetic age acceleration also yielded similar results (Additional file 1: Figure S9 and S10). Overall, the results were similar when using the last menstrual period-based estimate of gestational length instead of ultrasound measurements, both when looking at mean differences in gestational length and standardized birthweight, in addition to differences in the risk of preterm birth, post-term birth, SGA, and LGA (Additional file 1: Figures S11 and S12). Notably, no association with paternal epigenetic age estimates and post-term birth was observed when we used a survival analysis, censoring births that did not start spontaneously (initiated by induction or a caesarean section) (Additional file 1: Figure S13).

There was weak evidence of an interaction effect of chronological age on the relationship between maternal epigenetic age acceleration and gestational length for the PCGrimAge [mean difference − 0.12 days [95% CI − 0.87, 0.63] per SD increase for those younger than 30 vs. − 1.04 days (− 1.70, − 0.37) per SD increase for those ≥ 30 years of age; *p*-value for interaction 0.074] and DunedinPACE [mean difference − 0.24 days [95% CI − 1.02, 0.54] per SD increase for those younger than 30 vs. − 1.15 days (− 1.82, − 0.47) per SD increase for those ≥ 30 years of age; *p*-value for interaction 0.084] clocks (Additional file 1: Table S7). There was also evidence of an interaction with chronological age for the relationship between maternal epigenetic age acceleration and risk of spontaneous preterm birth for the PCGrimAge clock [adjusted OR 1.09 [95% CI 0.80, 1.49] per SD increase for those younger than 30 vs. 1.74 (95% CI 1.28, 2.37) per SD increase for those ≥ 30 years of age; *p*-value for interaction 0.036], as shown in Additional file 1: Table S8.

There was no robust evidence of any non-linear relationship between parental epigenetic age acceleration and risk of adverse birth outcomes, except for the association between maternal epigenetic age acceleration according to the Levine clock and having a large-for-gestational age offspring, with an interaction *p*-value of 0.037 (Additional file 1: Tables S9 and S10). With regard to differences in the associations by offspring sex, there was some evidence of an interaction for the association between maternal epigenetic age acceleration according to the Horvath clock and gestational age where an association as only seen among male offspring (*p*-value interaction 0.017), and some indication of an interaction for the association between maternal epigenetic age acceleration according to the DunedinPACE clock and risk of large-for-gestational age offspring with an association only among female offspring (*p*-value interaction 0.027) (Additional file 1: Tables S11 and S12).

## Discussion

In this population-based pregnancy cohort, increasing maternal epigenetic age acceleration was associated with shorter gestational length and higher risk of spontaneous preterm birth. This was consistent across most of the epigenetic age clocks that were evaluated. A similar relationship with gestational length was not observed with paternal epigenetic age acceleration. No clear differences in fetal growth or risk of pre-eclampsia were seen according to maternal or paternal epigenetic age acceleration. We observed some difference in the association between maternal epigenetic acceleration and offspring gestational age according to offspring sex, although this findings was only observed for the Horvath clock, and therefore not consistent but warranting further investigation.

Our findings are in line with those of previous studies indicating that maternal epigenetic age acceleration might be associated with shorter gestational length and increased risk of preterm birth [13, 15, 17, 18]. One study of 77 women from the USA found a significant decrease in gestational length with increasing epigenetic age for four out of the 15 clocks evaluated (estimates for the difference in weeks ranging from 2.10 × 10 − 5 to 0.17) [17]. A study of 296 women from the Philippines reported a decrease of 0.15 days per standard deviation increase in a Leptin-based clock (*p*-value 0.009), while no strong evidence of any associations was seen with the other 14 clocks evaluated [18]. A US study of 163 women at high risk for spontaneous preterm birth reported weak statistical evidence that accelerated biological aging estimated by the PCGrimAge clock was associated with the risk of preterm birth [OR of 1.73 (95% CI 0.81, 3.69)] [13]. A study of 177 women found a decreased gestational length with increasing maternal epigenetic age estimated by the Horvath clock [15]. The one study that investigated the relationship between paternal epigenetic age estimated from sperm cells and the risk of birth outcomes indicated some evidence of a shorter gestational length with increasing values [19].

We further showed that the difference in gestational length and risk of spontaneous preterm birth according to maternal epigenetic age acceleration varied by chronological age. Specifically, we found that the increased risk of spontaneous birth with increasing epigenetic age acceleration was greater among mothers who were 30 years of age or older. This supports the notion that epigenetic age acceleration might have a greater impact on pregnancy outcomes if one is chronologically older. As previous studies have not investigated this hypothesis, further research to elucidate whether there is an interaction between chronological age and epigenetic age acceleration on the risk of adverse birth outcomes seems warranted.

Existing evidence regarding a difference in birthweight according to maternal epigenetic age is more conflicted than what is reported for gestational length. The previously mentioned study from the Philippines found no differences in birthweight according to any of the 15 different clocks evaluated [18], while the US study found a decreased birthweight with four out of 15 clocks evaluated [17], where both studies adjusted for gestational length. Finally, evidence from existing studies of maternal epigenetic age and risk of preeclampsia is mixed, with one study providing evidence of increased epigenetic age acceleration being associated with increased risk of preeclampsia in three out of seven clocks evaluated [16], while another study provided no evidence of such a difference with any of the clocks [14].

Which underlying biological processes might be reflected in the decreased gestational length with maternal epigenetic age acceleration is currently unclear. While differences in pregnancy outcomes by parental age could reflect the role of aging on placentation, vascular dysfunction, genetic quality of the oocyte/sperm in aging parents, among other factors [1–4], differences according to epigenetic age acceleration could potentially reflect parental metabolic and physiological states [7, 8, 10–12]. Overall, the pattern of a decreased gestational length with increasing maternal epigenetic age acceleration was consistent across both first-generation clocks developed to predict chronological age (e.g., Horvath) and the second-generation clocks developed to predict diseases and biological risk factors (e.g., PCGrimAge). Several clinical biomarkers incorporated into some of the newer epigenetic clocks are associated with shorter gestational length and an increased risk of preterm birth, including maternal glucose levels/diabetes [28, 29], creatinine levels/kidney function [30, 31], and C-reactive protein (CRP) [32]. Therefore, it is plausible that the observed associations with shorter gestational length and increased risk of preterm birth may, at least in part, reflect the clinical components included in the development of the second-generation epigenetic age clocks. We did not have any clinical/blood measurements available, such as cholesterol levels, glucose levels, etc., and were therefore unable to explore how such values might be reflected the observed association between maternal epigenetic age acceleration and offspring gestational age. We did not observe an association between paternal epigenetic age acceleration and the risk of adverse pregnancy outcomes. The potential explanations for this remain speculative, but it may suggest that maternal epigenetic age acceleration has a stronger influence on placental and fetal development than paternal epigenetic age acceleration.

Until our findings are replicated in other large cohorts, it would be premature to propose that maternal epigenetic age acceleration measured during pregnancy can be used as a predictor of delivery outcomes in a clinical context, or to say that specific lifestyle interventions can be formulated to counteract epigenetic age acceleration and reduce the risk of adverse birth outcomes. Additional studies are therefore necessary to confirm our findings and provide more foundational evidence to enable more robust investigations into the potential biological mechanisms underlying the observed association between maternal epigenetic age acceleration and shorter gestational age and increased risk of spontaneous preterm birth.

Important strengths of this study are the population-based nature of the cohort, its sample size, and our ability to adjust for numerous relevant background characteristics. In addition, we examined multiple different epigenetic age clocks, providing a more comprehensive assessment. The estimates of maternal epigenetic age acceleration estimates may potentially be influenced by changes occurring during pregnancy [33], as the blood samples were taken around mid-pregnancy. However, it is unlikely that pregnancy influenced the paternal epigenetic age acceleration estimates. We also cannot exclude a possible selection bias, due to the participation rate in MoBa, which is reflected in the lower rate of some adverse birth outcomes when compared to the general population [34]. No added risk of selection bias was introduced by the sampling strategy used to obtain DNA methylation measurements, as our sample population is based on a random selection of eligible participants in the cohort. Statistical power was somewhat limited, as indicated by the wide confidence intervals for some of the estimates presented. Future studies with larger sample sizes should consider including more rare outcomes in relation to parental epigenetic age (such as stillbirth and congenital malformations). There is an element of multiple testing to our results, as we evaluated seven different epigenetic age acceleration estimates and multiple outcomes. Our results were not robust to Bonferroni correction for multiple testing, and require replication in larger sample sizes. Finally, our findings may be mostly generalizable to European-ancestry populations and may not apply to other ethnicities. Based on genotyping conducted on the majority of MoBa participants, approximately 95% of participants are of European ancestry [35].

## Conclusions

We found associations between maternal epigenetic age acceleration and shorter gestational length and increased risk of spontaneous preterm birth. These associations were consistent across multiple epigenetic age clocks. No clear associations were observed between paternal epigenetic age acceleration and any of the assessed birth outcomes. Further research is needed to confirm these findings, explore underlying biological mechanisms, and assess the generalizability of the results to more ethnically diverse populations.

## Supplementary Information

Additional file 1. Supplemental results, including Tables S1-S12 and Fig. S1-S13. Table S1. Background characteristics in eligible population of MoBa pregnancies. Table S2. Evaluation of the predictive value of the epigenetic age estimates. Table S3. Pearson correlation between epigenetic age acceleration estimates among mothers. Table S4. Correlation between epigenetic age acceleration estimates among fathers. Table S5. Correlation between epigenetic age acceleration estimates between partners. Fig S1. Smoothed plots of gestational length according to maternal epigenetic age acceleration. Fig S2. Smoothed plots of gestational length according to paternal epigenetic age acceleration. Fig S3. Smoothed plots of standardized birthweight according to maternal epigenetic age acceleration. Fig S4. Smoothed plots standardized birthweight according to paternal epigenetic age acceleration. Table S6. Distribution of adverse birth outcomes. Fig S5. Mean difference in gestational length and standardized birthweight according to parental epigenetic age acceleration with adjustment for cell type composition and gestational week of blood sampling. Fig S6. Risk of adverse birth outcomes according to parental epigenetic age acceleration with adjustment for cell type composition and gestational week of blood sampling. Fig S7. Mean difference in gestational length and standardized birthweight according to parental epigenetic age acceleration with adjustment for parity, education, smoking, body-mass index, diabetes and chronic hypertension. Fig S8. Risk of adverse birth outcomes according to parental epigenetic age acceleration for parity, education, smoking, body-mass index, diabetes and chronic hypertension. Fig S9. Mean difference in gestational length and standardized birthweight according to parental epigenetic age acceleration with adjustment for partner’s epigenetic age acceleration. Fig S10. Risk of adverse birth outcomes according to parental epigenetic age acceleration with adjustment for partner’s epigenetic age acceleration. Fig S11. Mean difference in gestational length and standardized birthweight according to parental epigenetic age acceleration using last menstrual period instead of ultrasound estimated gestational length. Fig S12. Risk of adverse birth outcomes according to parental epigenetic age acceleration using last menstrual period instead of ultrasound estimated gestational length. Fig S13. Risk of post-term birth according to parental epigenetic age acceleration using a survival analysis approach. Table S7. Differences in standardized birthweight and gestational age according to epigenetic age acceleration stratified by chronological age. Table S8. Differences in the odds of adverse birth outcomes according to parental epigenetic age acceleration stratified by chronological age. Table S9. Differences in standardized birthweight and gestational age according to categories of epigenetic age acceleration. Table S10. Differences in the odds of adverse birth outcomes according to categories of epigenetic age acceleration. Table S11. Differences in standardized birthweight and gestational age stratified by offspring sex. Table S12. Differences in the odds of adverse birth outcomes stratified by offspring sex.

## Data Availability

Data are not publicly available due to their containing information that could compromise the privacy of research participants. Summary data can be made available from the corresponding author on reasonable request.

## Abbreviations

CI: Confidence interval
LGA: Large for gestational age
MoBa: The Norwegian Mother, Father and Child Cohort Study
OR: Odds ratio
SD: Standard deviation
SGA: Small for gestational age
