# Correction: Parental epigenetic age acceleration and risk of adverse birth outcomes: the Norwegian mother, father and child cohort study

**DOI:** 10.1186/s12916-024-03824-y

**Published:** 2025-01-06

**Authors:** Maria C. Magnus, Yunsung Lee, Ellen Ø. Carlsen, Lise A. Arge, Astanand Jugessur, Liv G. Kvalvik, Nils-Halvdan Morken, Cecilia H. Ramlau-Hansen, Mikko Myrskylä, Per Magnus, Siri E. Håberg

**Affiliations:** 1 Centre for Fertility and Health, Norwegian Institute of Public Health, P.O. Box 222, Skøyen, Oslo, 0213 Norway; 2 Department of Global Public Health and Primary Care, University of Bergen, Bergen, Norway; 3 Department of Obstetrics and Gynecology, Haukeland University Hospital, Bergen, Norway; 4 Department of Public Health, Research Unit for Epidemiology, Aarhus University, Aarhus, Denmark; 5 Max Planck Institute for Demographic Research, Rostock, Germany; 6 Helsinki Institute for Demography and Population Health, University of Helsinki, Helsinki, Finland; 7 Max Planck, University of Helsinki Center for Social Inequalities in Population Health, Rostock, Germany

**Correction: BMC Med 22, 554 (2024)**

**https://doi.org/10.1186/s12916-024–03780-7**

The original article [1] initially erroneously presented co-author, Mikko Myrskylä’s name as Miko Myrskala; this has since been amended.
