# Supplementary material for: Parental epigenetic age acceleration and risk of adverse birth outcomes: the Norwegian mother, father and child cohort study
Source: BMC Med. 2024 Nov 25;22:554. doi: 10.1186/s12916-024-03780-7 (PMC11590542; doi:10.1186/s12916-024-03780-7)
Supplement: Supplementary file 1 — Additional file 1. Supplemental results, including Tables S1-S12 and Fig. S1-S13. Table S1. Background characteristics in eligible population of MoBa pregnancies. Table S2. Evaluation of the predictive value of the epigenetic age estimates. Table S3. Pearson correlation between epigenetic age acceleration estimates among mothers. Table S4. Correlation between epigenetic age acceleration estimates among fathers. Table S5. Correlation between epigenetic age acceleration estimates between partners. Fig S1. Smoothed plots of gestational length according to maternal epigenetic age acceleration. Fig S2. Smoothed plots of gestational length according to paternal epigenetic age acceleration. Fig S3. Smoothed plots of standardized birthweight according to maternal epigenetic age acceleration. Fig S4. Smoothed plots standardized birthweight according to paternal epigenetic age acceleration. Table S6. Distribution of adverse birth outcomes. Fig S5. Mean difference in gestational length and standardized birthweight according to parental epigenetic age acceleration with adjustment for cell type composition and gestational week of blood sampling. Fig S6. Risk of adverse birth outcomes according to parental epigenetic age acceleration with adjustment for cell type composition and gestational week of blood sampling. Fig S7. Mean difference in gestational length and standardized birthweight according to parental epigenetic age acceleration with adjustment for parity, education, smoking, body-mass index, diabetes and chronic hypertension. Fig S8. Risk of adverse birth outcomes according to parental epigenetic age acceleration for parity, education, smoking, body-mass index, diabetes and chronic hypertension. Fig S9. Mean difference in gestational length and standardized birthweight according to parental epigenetic age acceleration with adjustment for partner’s epigenetic age acceleration. Fig S10. Risk of adverse birth outcomes according to parental epigenetic age acceleration with a [file 12916_2024_3780_MOESM1_ESM.docx]

Table S1 Background characteristics in eligible population of MoBa pregnancies

| **Background characteristics** | **Pregnancies eligible for analysis of maternal epigenetic age**  **(n=98,161)** | **Pregnancies eligible for analysis of paternal epigenetic age**  **(n=73,509)** |
| --- | --- | --- |
| **Age at delivery, mean(SD)** | 30.1 (4.6) | 32.6 (5.3) |
| **Educational level, n(%)** |  |  |
| Less than high-school | 5,998 (6.1) | 5,491 (7.5) |
| High-school | 26,707 (27.2) | 27,046 (36.8) |
| College, up to 4 years | 40,344 (41.1) | 20,370 (27.7) |
| College, more than 4 years | 24,609 (25.1) | 18,372 (24.9) |
| Missing | 503 (0.5) | 2,230 (3.0) |
| **Primiparous, n(%)** |  |  |
| No | 54,463 (55.5) | NA |
| Yes | 43,256 (44.2) | NA |
| Missing | 442 (0.5 |  |
| **Smoking status around the start of pregnancy, n(%)*** |  |  |
| Never | 48,668 (49.58) | 34,806 (47.4) |
| Former | 20,155 (20.5) | 15,670 (21.3) |
| Current | 27,782 (28.3) | 22,422 (30.5) |
| Missing | 1,556 (1.6) | 611 (0.8) |
| **Body-mass index , n(%)** |  |  |
| <25 | 65,433 (66.7) | 31,513 (42.9) |
| 25-29.9 | 20,827 (21.2) | 31,909 (43.4) |
| ≥30 | 9,122 (9.3) | 7,140 (9.7) |
| Missing | 2,779 (2.8) | 2,947 (4.0) |
| **Diabetes mellitus, n(%)** |  |  |
| No | 97,533 (99.4) | 72,861 (99.1) |
| Yes | 628 (0.6) | 648 (0.9) |
| **Chronic hypertension, n(%)** |  |  |
| No | 97,127 (98.9) | 71,465 (97.2) |
| Yes | 1,034 (1.1) | 2,044 (2.8) |

*Reflects the last 3 months prior to pregnancy for mothers and the last 6 months prior to pregnancy for fathers.

Table S2 Evaluation of the predictive value of the epigenetic age estimates

| Epigentic clock | Mothers | | | Fathers | | |
| --- | --- | --- | --- | --- | --- | --- |
|  | Pearson correlation coefficient with chronological age | Mean absolute error | R^2^ | Pearson correlation coefficient with chronological age | Mean absolute error | R^2^ |
| Hannum | 0.75 | 8.67 | 0.57 | 0.67 | 11.27 | 0.61 |
| Horvath | 0.56 | 13.76 | 0.31 | 0.36 | 25.42 | 0.13 |
| Levine | 0.64 | 11.66 | 0.42 | 0.71 | 14.64 | 0.50 |
| DNAmTL | 0.46 | 15.64 | 0.22 | 0.56 | 20.22 | 0.31 |
| PCGrimage | 0.76 | 8.44 | 0.58 | 0.79 | 10.90 | 0.63 |

Table S3 Pearson correlation between epigenetic age acceleration estimates among mothers

| Epigentic clock | Hannum | Horvath | Levine | Dunedinpace | DNAmTL | PCgrimage |
| --- | --- | --- | --- | --- | --- | --- |
| Hannum | 1 |  |  |  |  |  |
| Horvath | 0.12 | 1 |  |  |  |  |
| Levine | 0.50 | 0.56 | 1 |  |  |  |
| DunedinPACE | 0.21 | 0.02 | 0.38 | 1 |  |  |
| DNAmTL | 0.42 | -0.09 | 0.26 | 0.23 | 1 |  |
| PCGrimage | 0.23 | 0.33 | 0.51 | 0.55 | 0.29 | 1 |

Table S4 Correlation between epigenetic age acceleration estimates among fathers

| Epigentic clock | Hannum | Horvath | Levine | Dunedinpace | DNAmTL | PCgrimage |
| --- | --- | --- | --- | --- | --- | --- |
| Hannum | 1 |  |  |  |  |  |
| Horvath | 0.10 | 1 |  |  |  |  |
| Levine | 0.46 | 0.59 | 1 |  |  |  |
| DunedinPACE | 0.16 | 0.02 | 0.31 | 1 |  |  |
| DNAmTL | 0.46 | -0.07 | 0.31 | 0.29 | 1 |  |
| PCGrimage | 0.19 | 0.30 | 0.51 | 0.61 | 0.28 | 1 |

Table S5 Correlation between epigenetic age acceleration estimates between partners

| Epigentic clock | Hannum | Horvath | Levine | Dunedinpace | DNAmTL | PCgrimage |
| --- | --- | --- | --- | --- | --- | --- |
| Hannum | 0.21 |  |  |  |  |  |
| Horvath |  | 0.67 |  |  |  |  |
| Levine |  |  | 0.26 |  |  |  |
| DunedinPACE |  |  |  | 0.13 |  |  |
| DNAmTL |  |  |  |  | 0.12 |  |
| PCGrimage |  |  |  |  |  | 0.32 |

Fig S1 Smoothed plots of gestational length according to maternal epigenetic age acceleration


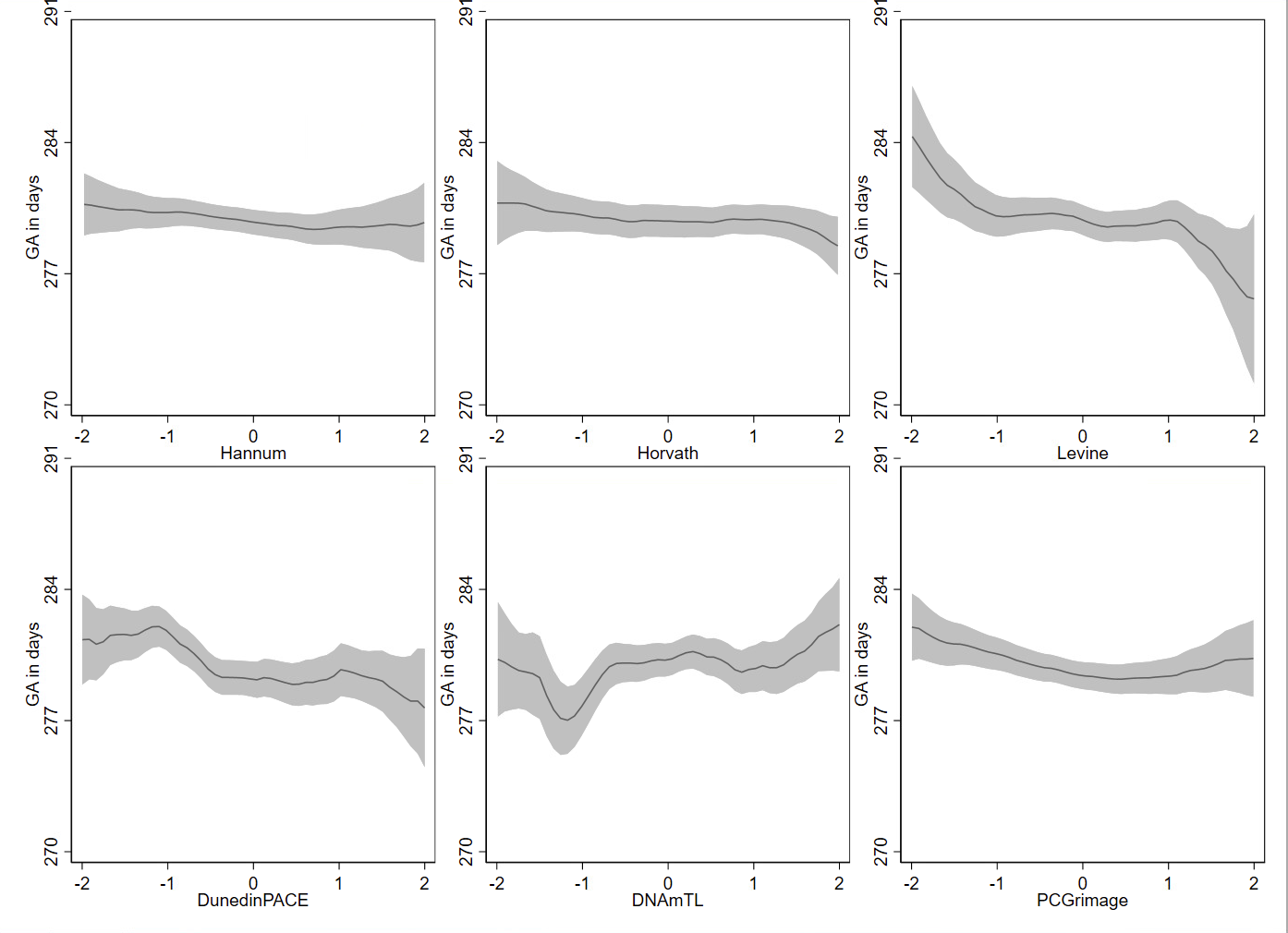


Fig S2 Smoothed plots of gestational length according to paternal epigenetic age acceleration


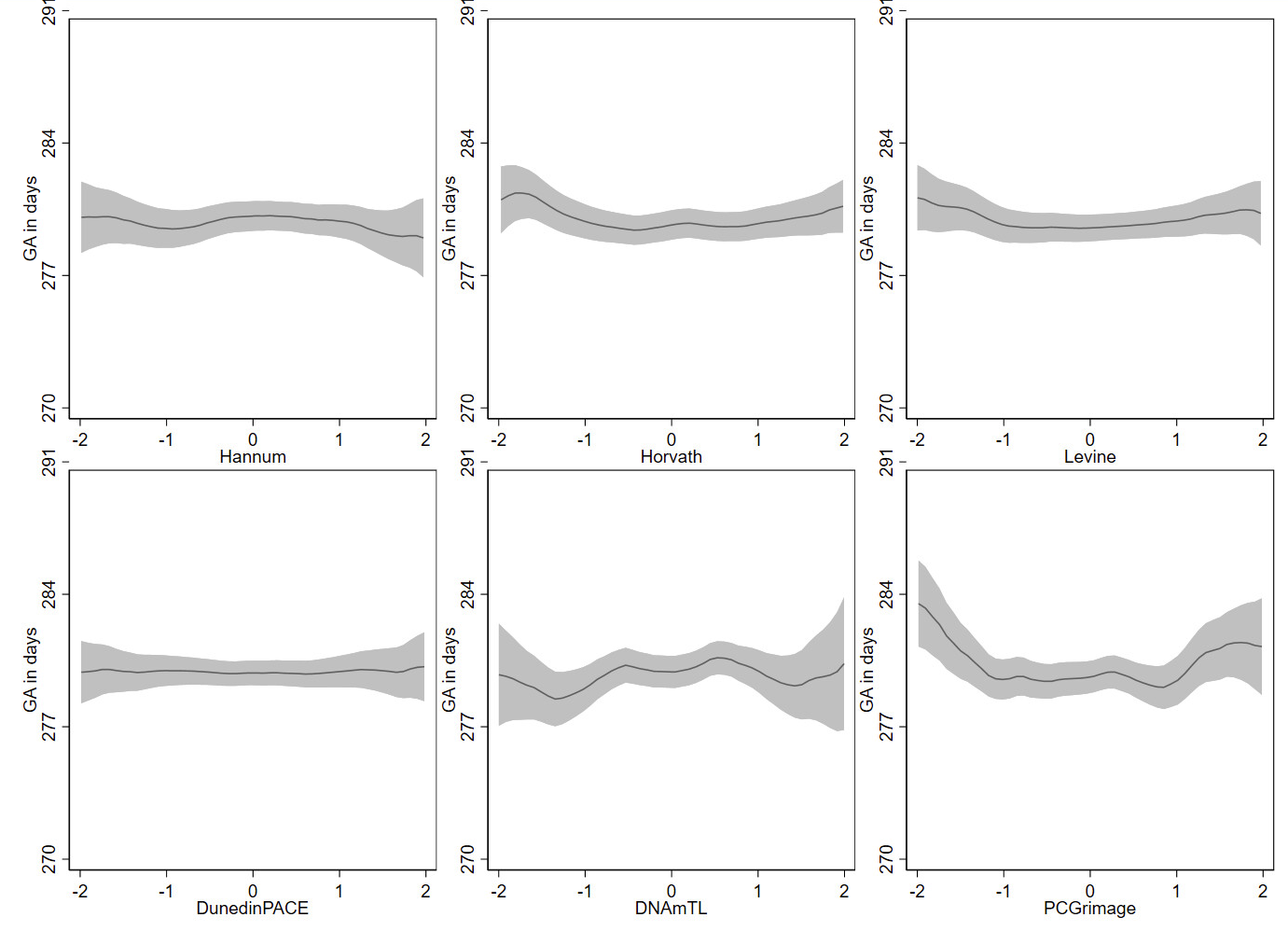


Fig S3 Smoothed plots of standardized birthweight according to maternal epigenetic age acceleration


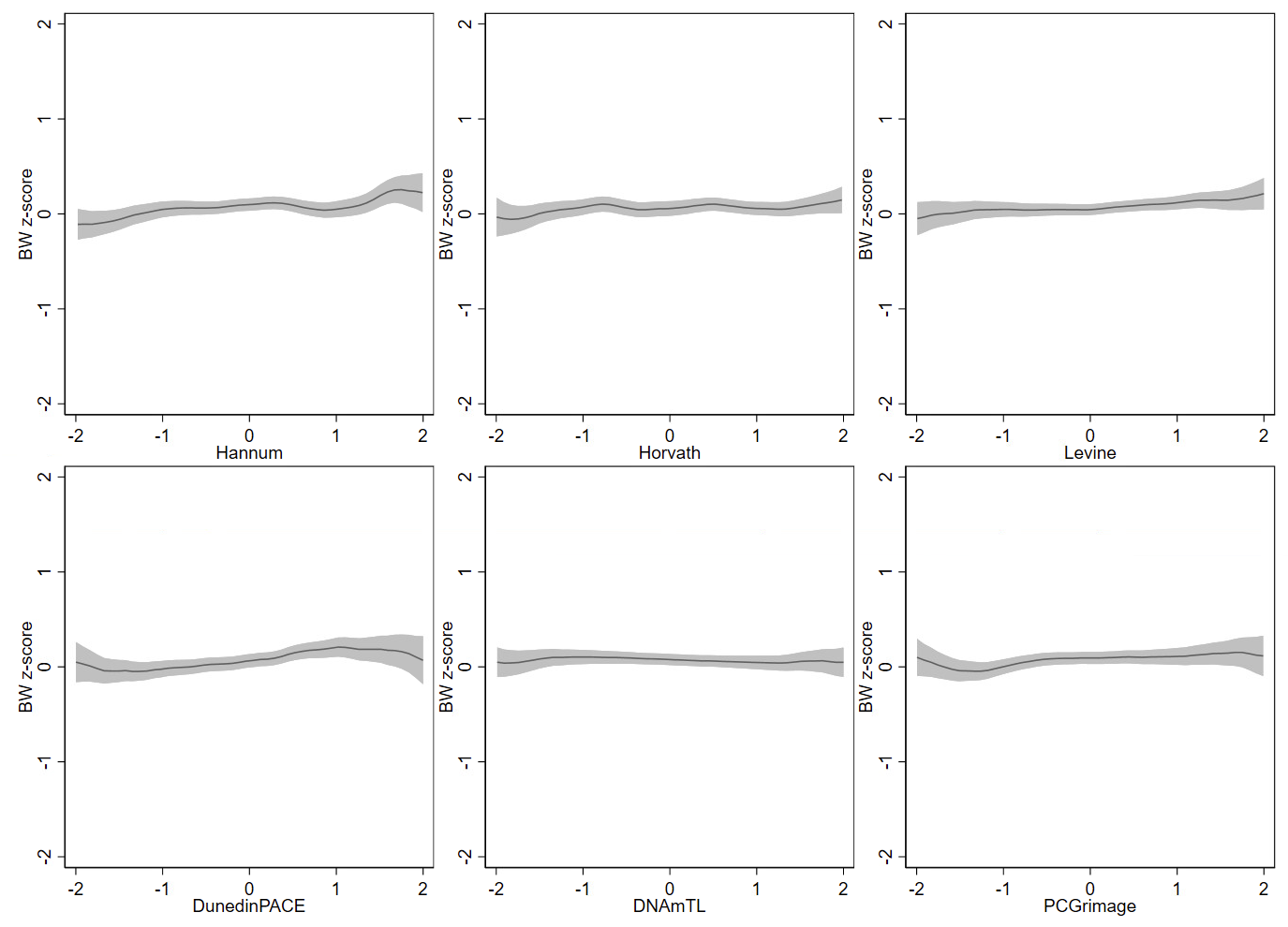


Fig S4 Smoothed plots standardized birthweight according to paternal epigenetic age acceleration


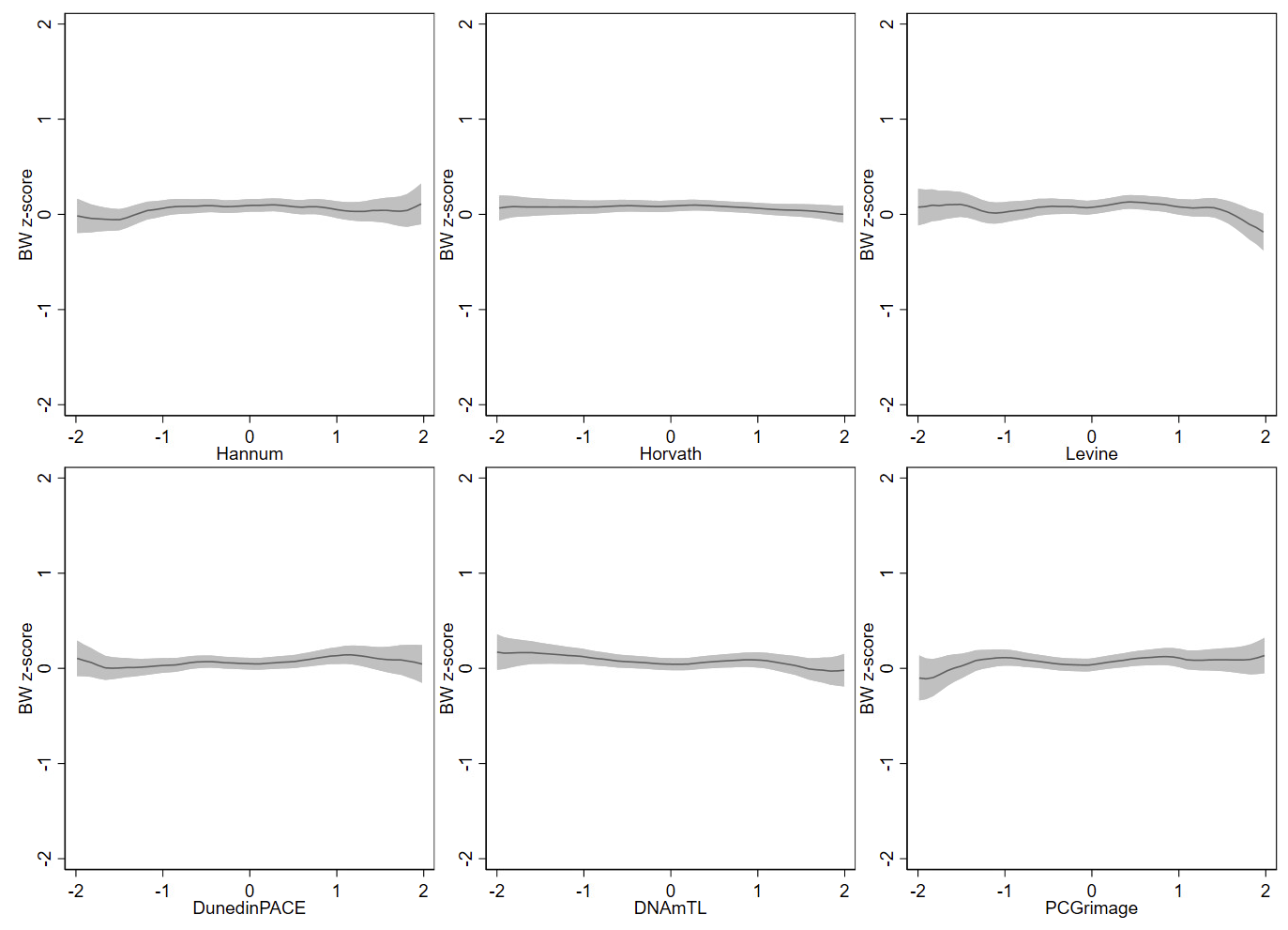


Table S6 Distribution of adverse birth outcomes

| Outcome | N(%) cases |
| --- | --- |
| Preterm birth | 104 (4.7) |
| Spontaneous preterm birth | 72 (3.3) |
| Induced preterm birth | 32 (1.5) |
| Post-term birth | 166 (7.6) |
| Small-for-gestational age | 179 (8.1) |
| Large-for-gestational age | 239 (10.9) |
| Pre-eclampsia | 69 (3.1) |

Fig S5 Mean difference in gestational length and standardized birthweight according to parental epigenetic age acceleration with adjustment for cell type composition and gestational week of blood sampling

Fig S6 Risk of adverse birth outcomes according to parental epigenetic age acceleration with adjustment for cell type composition and gestational week of blood sampling

Fig S7 Mean difference in gestational length and standardized birthweight according to parental epigenetic age acceleration with adjustment for parity, education, smoking, body-mass index, diabetes and chronic hypertension

Fig S8 Risk of adverse birth outcomes according to parental epigenetic age acceleration for parity, education, smoking, body-mass index, diabetes and chronic hypertension

Fig S9 Mean difference in gestational length and standardized birthweight according to parental epigenetic age acceleration with adjustment for partner’s epigenetic age acceleration

Fig S10 Risk of adverse birth outcomes according to parental epigenetic age acceleration with adjustment for partner’s epigenetic age acceleration

Fig S11 Mean difference in gestational length and standardized birthweight according to parental epigenetic age acceleration using last menstrual period instead of ultrasound estimated gestational length

Fig S12 Risk of adverse birth outcomes according to parental epigenetic age acceleration using last menstrual period instead of ultrasound estimated gestational length

Fig S13 Risk of post-term birth according to parental epigenetic age acceleration using a survival analysis approach

Table S7 Differences in standardized birthweight and gestational age according to epigenetic age acceleration stratified by chronological age

| Outcome | Epigenetic age clock | Mothers | | | Fathers | | |
| --- | --- | --- | --- | --- | --- | --- | --- |
|  |  | <30 years  Unadjusted mean difference (95% CI)  (N=1,093) | ≥30 years  Unadjusted mean difference (95% CI)  (N=1,105) | p-value for interaction^a^ | <30 years  Unadjusted mean difference (95% CI)  (N=715) | ≥30 years  Unadjusted mean difference (95% CI)  (N=1,478) | p-value for interaction^a^ |
| Gestational age in days | Hannum | -0.71 (-1.47,0.06) | 0.04 (-0.60,0.68) | 0.139 | -0.61 (-1.81,0.59) | -0.45 (-1.69,0.80) | 0.438 |
|  | Horvath | -0.53 (-1.26,0.20) | -0.54 (-1.21,0.13) | 0.981 | -0.32 (-1.69,1.06) | -0.24 (-1.44,0.97) | 0.943 |
|  | Levine | -0.73 (-1.48,0.02) | -0.87 (-1.52,-0.22) | 0.787 | 0.59 (-0.72,1.90) | 0.55 (-0.61,1.71) | 0.612 |
|  | DunedinPACE | -0.24 (-1.02,0.54) | -1.15 (-1.82,-0.47) | 0.084 | 0.25 (-0.95,1.45) | 0.23 (-1.01,1.48) | 0.357 |
|  | DNAmTL | 0.70 (-0.06,1.46) | 0.50 (-0.16,1.15) | 0.692 | -0.61 (-1.86,0.64) | 0.18 (-1.03,1.38) | 0.054 |
|  | PCGrimAge | -0.12 (-0.87,0.63) | -1.04 (-1.70,-0.37) | 0.074 | 0.42 (-0.81,1.64) | 0.24 (-0.98,1.46) | 0.056 |
| Standardized birthweight | Hannum | 0.03 (-0.03,0.08) | 0.05 (-0.01,0.10) | 0.653 | -0.05 (-0.15,0.05) | -0.03 (-0.13,0.07) | 0.747 |
|  | Horvath | 0.01 (-0.05,0.06) | 0.01 (-0.05,0.07) | 0.987 | -0.03 (-0.14,0.08) | -0.04 (-0.14,0.06) | 0.797 |
|  | Levine | 0.04 (-0.01,0.10) | 0.03 (-0.03,0.08) | 0.688 | -0.02 (-0.13,0.08) | -0.02 (-0.11,0.08) | 0.653 |
|  | DunedinPACE | 0.08 (0.02,0.14) | 0.09 (0.03,0.15) | 0.734 | 0.04 (-0.06,0.13) | 0.12 (0.02,0.23) | 0.838 |
|  | DNAmTL | -0.00 (-0.06,0.05) | -0.03 (-0.09,0.03) | 0.521 | 0.08 (-0.03,0.18) | 0.04 (-0.06,0.14) | 0.441 |
|  | PCGrimAge | 0.01 (-0.05,0.07) | 0.04 (-0.02,0.10) | 0.568 | 0.05 (-0.05,0.15) | 0.07 (-0.03,0.17) | 0.643 |

^a^Test for interaction conducted by adding a product term between a binary indicator for chronological age (above or below 30) with the continuous measure of epigenetic age acceleration.

Table S8 Differences in the odds of adverse birth outcomes according to parental epigenetic age acceleration stratified by chronological age

| Outcome | Epigenetic age clock | Mothers | | | | | Fathers | | | | |
| --- | --- | --- | --- | --- | --- | --- | --- | --- | --- | --- | --- |
|  |  | <30 years | | ≥30 years | | p-value for interaction^a^ | <30 years | | ≥30 years | | p-value for interaction^a^ |
|  |  | N (n cases) | OR (95% CI) | N (n cases) | OR (95% CI) |  | N (n cases) | OR (95% CI) | N (n cases) | OR (95% CI) |  |
| Preterm birth | Hannum | 1021 (56) | 0.96 (0.73,1.26) | 1004 (48) | 0.88 (0.70,1.10) | 0.628 | 649 (41) | 1.01 (0.73,1.39) | 1370 (63) | 1.03 (0.81,1.32) | 0.916 |
|  | Horvath | 1021 (56) | 1.11 (0.85,1.44) | 1004 (48) | 1.17 (0.87,1.58) | 0.783 | 649 (41) | 1.03 (0.75,1.42) | 1370 (63) | 1.04 (0.81,1.33) | 0.986 |
|  | Levine | 1021 (56) | 1.15 (0.87,1.53) | 1004 (48) | 1.35 (0.98,1.87) | 0.472 | 649 (41) | 0.97 (0.70,1.36) | 1370 (63) | 1.05 (0.82,1.36) | 0.717 |
|  | DunedinPACE | 1021 (56) | 1.00 (0.76,1.32) | 1004 (48) | 1.25 (0.93,1.68) | 0.283 | 649 (41) | 0.92 (0.66,1.27) | 1370 (63) | 1.14 (0.89,1.47) | 0.297 |
|  | DNAmTL | 1021 (56) | 0.90 (0.69,1.18) | 1004 (48) | 0.85 (0.65,1.13) | 0.779 | 649 (41) | 1.26 (0.92,1.72) | 1370 (63) | 0.85 (0.66,1.09) | 0.057 |
|  | PCGrimAge | 1021 (56) | 0.95 (0.72,1.26) | 1004 (48) | 1.38 (1.06,1.80) | 0.061 | 649 (41) | 1.02 (0.72,1.43) | 1370 (63) | 1.18 (0.93,1.50) | 0.474 |
| Spontaneous preterm birth | Hannum | 1006 (41) | 0.99 (0.72,1.36) | 987 (31) | 1.00 (0.72,1.40) | 0.960 | 638 (309 | 1.00 (0.69,1.44) | 1349 (42) | 0.98 (0.74,1.31) | 0.948 |
|  | Horvath | 1006 (41) | 1.04 (0.77,1.42) | 987 (31) | 1.37 (0.94,2.01) | 0.274 | 638 (309 | 1.04 (0.72,1.50) | 1349 (42) | 1.06 (0.78,1.44) | 0.936 |
|  | Levine | 1006 (41) | 1.19 (0.86,1.66) | 987 (31) | 1.92 (1.26,2.91) | 0.082 | 638 (309 | 0.91 (0.62,1.33) | 1349 (42) | 1.18 (0.86,1.63) | 0.301 |
|  | DunedinPACE | 1006 (41) | 1.09 (0.79,1.51) | 987 (31) | 1.34 (0.93,1.93) | 0.400 | 638 (309 | 0.90 (0.61,1.31) | 1349 (42) | 1.29 (0.96,1.73) | 0.138 |
|  | DNAmTL | 1006 (41) | 0.81 (0.60,1.11) | 987 (31) | 0.80 (0.57,1.12) | 0.936 | 638 (309 | 1.18 (0.82,1.70) | 1349 (42) | 0.93 (0.68,1.26) | 0.315 |
|  | PCGrimAge | 1006 (41) | 1.09 (0.80,1.49) | 987 (31) | 1.74 (1.28,2.37) | 0.036 | 638 (309 | 1.03 (0.69,1.53) | 1349 (42) | 1.25 (0.94,1.66) | 0.439 |
| Induced preterm birth | Hannum | 980 (15) | 0.88 (0.53,1.45) | 973 (17) | 0.78 (0.60,1.01) | 0.673 | 619 (11) | 1.04 (0.57,1.92) | 1328 (21) | 1.14 (0.75,1.75) | 0.808 |
|  | Horvath | 980 (15) | 1.30 (0.78,2.19) | 980 (15) | 0.90 (0.57,1.43) | 0.294 | 619 (11) | 1.02 (0.56,1.85) | 1328 (21) | 1.00 (0.65,1.52) | 0.950 |
|  | Levine | 980 (15) | 1.05 (0.62,1.78) | 980 (15) | 0.87 (0.64,1.18) | 0.545 | 619 (11) | 1.18 (0.62,2.23) | 1328 (21) | 0.87 (0.61,1.24) | 0.413 |
|  | DunedinPACE | 980 (15) | 0.78 (0.46,1.33) | 980 (15) | 1.08 (0.66,1.77) | 0.381 | 619 (11) | 0.98 (0.53,1.80) | 1328 (21) | 0.87 (0.56,1.37) | 0.768 |
|  | DNAmTL | 980 (15) | 1.20 (0.71,2.05) | 980 (15) | 0.96 (0.60,1.53) | 0.533 | 619 (11) | 1.50 (0.81,2.78) | 1328 (21) | 0.72 (0.48,1.09) | 0.051 |
|  | PCGrimAge | 980 (15) | 0.65 (0.38,1.12) | 980 (15) | 0.80 (0.49,1.32) | 0.578 | 619 (11) | 0.98 (0.51,1.87) | 1328 (21) | 1.06 (0.69,1.61) | 0.846 |
| Post-term | Hannum | 1034 (69) | 0.93 (0.73,1.19) | 1053 (97) | 0.93 (0.77,1.11) | 0.984 | 671 (63) | 1.22 (0.93,1.60) | 1411 (104) | 1.00 (0.83,1.21) | 0.236 |
|  | Horvath | 1034 (69) | 1.12 (0.88,1.43) | 1053 (97) | 0.97 (0.79,1.19) | 0.359 | 671 (63) | 1.29 (0.99,1.69) | 1411 (104) | 1.15 (0.94,1.41) | 0.499 |
|  | Levine | 1034 (69) | 0.98 (0.77,1.24) | 1053 (97) | 0.90 (0.76,1.07) | 0.587 | 671 (63) | 1.07 (0.82,1.41) | 1411 (104) | 1.28 (1.03,1.58) | 0.320 |
|  | DunedinPACE | 1034 (69) | 1.08 (0.84,1.40) | 1053 (97) | 0.83 (0.68,1.02) | 0.111 | 671 (63) | 1.08 (0.83,1.39) | 1411 (104) | 1.09 (0.89,1.33) | 0.926 |
|  | DNAmTL | 1034 (69) | 0.95 (0.74,1.22) | 1053 (97) | 1.17 (0.96,1.44) | 0.192 | 671 (63) | 1.08 (0.83,1.39) | 1411 (104) | 0.88 (0.72,1.07) | 0.220 |
|  | PCGrimAge | 1034 (69) | 1.20 (0.95,1.52) | 1053 (97) | 0.94 (0.76,1.16) | 0.128 | 671 (63) | 1.23 (0.95,1.61) | 1411 (104) | 1.34 (1.11,1.61) | 0.627 |
| sga | Hannum | 982 (86) | 0.96 (0.76,1.20) | 976 (93) | 0.79 (0.63,0.98) | 0.226 | 640 (53) | 0.93 (0.70,1.24) | 1314 (127) | 1.03 (0.86,1.23) | 0.555 |
|  | Horvath | 982 (86) | 0.95 (0.76,1.18) | 976 (93) | 0.90 (0.72,1.11) | 0.724 | 640 (53) | 1.03 (0.78,1.37) | 1314 (127) | 1.01 (0.84,1.21) | 0.888 |
|  | Levine | 982 (86) | 0.93 (0.74,1.18) | 976 (93) | 0.83 (0.66,1.04) | 0.484 | 640 (53) | 1.02 (0.76,1.36) | 1314 (127) | 0.98 (0.82,1.18) | 0.862 |
|  | DunedinPACE | 982 (86) | 1.22 (0.97,1.55) | 976 (93) | 0.95 (0.77,1.18) | 0.127 | 640 (53) | 0.87 (0.65,1.15) | 1314 (127) | 0.93 (0.77,1.13) | 0.667 |
|  | DNAmTL | 982 (86) | 0.92 (0.73,1.15) | 976 (93) | 1.15 (0.94,1.42) | 0.140 | 640 (53) | 1.16 (0.88,1.54) | 1314 (127) | 0.94 (0.78,1.13) | 0.204 |
|  | PCGrimAge | 982 (86) | 1.18 (0.96,1.46) | 976 (93) | 0.86 (0.69,1.07) | 0.040 | 640 (53) | 0.84 (0.62,1.15) | 1314 (127) | 0.94 (0.78,1.13) | 0.555 |
| lga | Hannum | 1006 (110) | 1.01 (0.83,1.24) | 1012 (129) | 1.02 (0.85,1.22) | 0.964 | 640 (53) | 0.95 (0.74,1.21) | 1351 (164) | 0.95 (0.81,1.10) | 0.990 |
|  | Horvath | 1006 (110) | 1.09 (0.90,1.32) | 1006 (110) | 1.01 (0.84,1.21) | 0.563 | 640 (53) | 1.13 (0.88,1.45) | 1351 (164) | 0.93 (0.80,1.09) | 0.220 |
|  | Levine | 1006 (110) | 1.12 (0.91,1.37) | 1006 (110) | 0.99 (0.83,1.18) | 0.360 | 640 (53) | 1.11 (0.86,1.44) | 1351 (164) | 0.91 (0.78,1.05) | 0.186 |
|  | DunedinPACE | 1006 (110) | 1.24 (1.01,1.52) | 1006 (110) | 1.18 (0.98,1.43) | 0.767 | 640 (53) | 0.98 (0.77,1.25) | 1351 (164) | 1.07 (0.91,1.26) | 0.575 |
|  | DNAmTL | 1006 (110) | 0.97 (0.80,1.19) | 1006 (110) | 1.03 (0.86,1.23) | 0.685 | 640 (53) | 1.08 (0.85,1.37) | 1351 (164) | 1.08 (0.91,1.27) | 0.965 |
|  | PCGrimAge | 1006 (110) | 1.11 (0.91,1.35) | 1006 (110) | 1.03 (0.86,1.24) | 0.600 | 640 (53) | 0.84 (0.64,1.10) | 1351 (164) | 0.89 (0.75,1.05) | 0.739 |
| preeclampsia | Hannum | 1093 (32) | 1.27 (0.88,1.83) | 1105 (37) | 1.00 (0.73,1.36) | 0.613 | 715 (27) | 1.19 (0.80,1.78) | 1478 (42) | 0.96 (0.72,1.28) | 0.392 |
|  | Horvath | 1351 (164) | 1.01 (0.71,1.42) | 1105 (37) | 1.22 (0.87,1.71) | 0.423 | 715 (27) | 1.40 (0.93,2.12) | 1478 (42) | 0.94 (0.70,1.27) | 0.125 |
|  | Levine | 1351 (164) | 1.31 (0.90,1.91) | 1105 (37) | 1.06 (0.75,1.48) | 0.281 | 715 (27) | 1.75 (1.14,2.69) | 1478 (42) | 0.96 (0.72,1.29) | 0.024 |
|  | DunedinPACE | 1351 (164) | 1.01 (0.70,1.46) | 1105 (37) | 1.26 (0.91,1.75) | 0.948 | 715 (27) | 1.05 (0.72,1.54) | 1478 (42) | 1.03 (0.76,1.40) | 0.936 |
|  | DNAmTL | 1351 (164) | 0.87 (0.62,1.24) | 1105 (37) | 1.01 (0.74,1.39) | 0.644 | 715 (27) | 1.05 (0.72,1.53) | 1478 (42) | 1.09 (0.80,1.48) | 0.892 |
|  | PCGrimAge | 1351 (164) | 0.83 (0.58,1.19) | 1105 (37) | 1.06 (0.77,1.46) | 0.319 | 715 (27) | 1.32 (0.90,1.95) | 1478 (42) | 0.85 (0.62,1.17) | 0.085 |

^a^Test for interaction conducted by adding a product term between a binary indicator for chronological age (above or below 30) with the continuous measure of epigenetic age acceleration.

Table S9 Differences in standardized birthweight and gestational age according to categories of epigenetic age acceleration

| Outcome | Epigenetic age clock | Mothers | | | | | Fathers | | | | |
| --- | --- | --- | --- | --- | --- | --- | --- | --- | --- | --- | --- |
|  |  | Deceleration  (z-score <-0.5) | | Acceleration  (z-score >0.5) | | p-value for test of non-linear relationship^a^ | Deceleration  (z-score <-0.5) | | Acceleration (z-score >0.5) | | p-value for test of non-linear relationship^a^ |
|  |  | N | Mean difference (95% CI) | N | Mean difference (95% CI) |  | N | Mean difference (95% CI) | N | Mean difference (95% CI) |  |
| Gestational age in days  (2191 women; 2186 men) | Hannum | 698 | 0.54 (-0.66,1.73) | 608 | -0.36 (-1.61,0.88) | 0.729 | 701 | -0.61 (-1.81,0.59) | 619 | -0.45 (-1.69,0.80) | 0.409 |
|  | Horvath | 588 | 0.76 (-0.60,2.13) | 1001 | -0.25 (-1.47,0.97) | 0.985 | 555 | -0.32 (-1.69,1.06) | 1002 | -0.24 (-1.44,0.97) | 0.380 |
|  | Levine | 572 | 0.90 (-0.36,2.16) | 726 | -0.46 (-1.64,0.72) | 0.300 | 519 | 0.59 (-0.72,1.90) | 793 | 0.55 (-0.61,1.71) | 0.643 |
|  | DunedinPACE | 693 | 1.66 (0.46,2.86) | 629 | -0.34 (-1.57,0.89) | 0.105 | 678 | 0.25 (-0.95,1.45) | 599 | 0.23 (-1.01,1.48) | 0.986 |
|  | DNAmTL | 648 | -1.65 (-2.88,-0.42) | 708 | -0.51 (-1.71,0.69) | 0.768 | 630 | -0.61 (-1.86,0.64) | 726 | 0.18 (-1.03,1.38) | 0.713 |
|  | PCGrimAge | 645 | 1.51 (0.29,2.73) | 668 | 0.20 (-1.01,1.41) | 0.160 | 618 | 0.42 (-0.81,1.64) | 619 | 0.24 (-0.98,1.46) | 0.147 |
| Standardized birthweight  (2189 women; 2184 men) | Hannum | 698 | -0.06 (-0.16,0.04) | 607 | 0.01 (-0.09,0.11) | 0.024 | 700 | -0.05 (-0.15,0.05) | 619 | -0.03 (-0.13,0.07) | 0.221 |
|  | Horvath | 588 | -0.01 (-0.12,0.10) | 999 | 0.01 (-0.09,0.11) | 0.111 | 555 | -0.03 (-0.14,0.08) | 1000 | -0.04 (-0.14,0.06) | 0.853 |
|  | Levine | 572 | -0.00 (-0.11,0.10) | 725 | 0.14 (0.04,0.24) | 0.006 | 518 | -0.02 (-0.13,0.08) | 793 | -0.02 (-0.11,0.08) | 0.739 |
|  | DunedinPACE | 693 | -0.09 (-0.19,0.01) | 627 | 0.13 (0.02,0.23) | 0.553 | 676 | 0.04 (-0.06,0.13) | 599 | 0.12 (0.02,0.23) | 0.689 |
|  | DNAmTL | 647 | 0.00 (-0.10,0.10) | 708 | -0.05 (-0.15,0.05) | 0.692 | 630 | 0.08 (-0.03,0.18) | 724 | 0.04 (-0.06,0.14) | 0.711 |
|  | PCGrimAge | 645 | -0.06 (-0.16,0.04) | 667 | 0.00 (-0.10,0.10) | 0.068 | 617 | 0.05 (-0.05,0.15) | 619 | 0.07 (-0.03,0.17) | 0.904 |

^a^ Test for non-linear relationship conducted by adding a second order term for the measures of epigenetic age acceleration in the model .

Table S10 Differences in the odds of adverse birth outcomes according to categories of epigenetic age acceleration

| Outcome | Epigenetic age clock | Mothers | | | | | Fathers | | | | |
| --- | --- | --- | --- | --- | --- | --- | --- | --- | --- | --- | --- |
|  |  | Deceleration  (z-score <-0.5) | | Acceleration  (z-score >-0.5) | | p-value for test of non-linear relationship^a^ | Deceleration  (z-score <-0.5) | | Acceleration  (z-score >-0.5) | | p-value for test of non-linear relationship^a^ |
|  |  | N (n cases) | OR (95% CI) | N (n cases) | OR (95% CI) |  | N (n cases) | OR (95% CI) | N (n cases) | OR (95% CI) |  |
| Preterm birth | Hannum | 639 (38) | 1.34 (0.84,2.14) | 563 (29) | 1.15 (0.70,1.90) | 0.415 | 652 (36) | 1.08 (0.68,1.70) | 571 (27) | 0.91 (0.56,1.50) | 0.521 |
|  | Horvath | 546 (24) | 0.80 (0.46,1.39) | 925 (50) | 1.00 (0.63,1.59) | 0.934 | 524 (30) | 1.48 (0.85,2.58) | 911 (51) | 1.45 (0.87,2.39) | 0.426 |
|  | Levine | 521 (22) | 0.78 (0.46,1.31) | 667 (37) | 1.03 (0.66,1.62) | 0.767 | 485 (25) | 1.00 (0.60,1.65) | 723 (37) | 0.99 (0.63,1.55) | 0.376 |
|  | DunedinPACE | 633 (28) | 0.77 (0.47,1.24) | 582 (30) | 0.90 (0.56,1.45) | 0.382 | 627 (33) | 1.12 (0.70,1.79) | 547 (31) | 1.21 (0.75,1.96) | 0.396 |
|  | DNAmTL | 607 (39) | 1.53 (0.95,2.47) | 649 (32) | 1.16 (0.70,1.90) | 0.794 | 579 (33) | 1.23 (0.76,2.00) | 670 (35) | 1.12 (0.70,1.81) | 0.458 |
|  | PCGrimAge | 600 (25) | 0.71 (0.43,1.16) | 613 (32) | 0.90 (0.56,1.42) | 0.364 | 586 (26) | 0.90 (0.55,1.48) | 557 (35) | 1.30 (0.82,2.06) | 0.374 |
| Spontaneous preterm birth | Hannum | 622 (21) | 0.92 (0.52,1.62) | 555 (21) | 1.03 (0.58,1.82) | 0.460 | 645 (29) | 1.55 (0.88,2.70) | 564 (20) | 1.21 (0.66,2.22) | 0.655 |
|  | Horvath | 538 (16) | 0.85 (0.43,1.66) | 912 (37) | 1.17 (0.66,2.05) | 0.797 | 513 (19) | 1.27 (0.65,2.47) | 896 (36) | 1.38 (0.77,2.48) | 0.565 |
|  | Levine | 510 (11) | 0.55 (0.27,1.09) | 659 (29) | 1.14 (0.68,1.90) | 0.844 | 474 (14) | 0.75 (0.40,1.43) | 713 (27) | 0.98 (0.58,1.65) | 0.666 |
|  | DunedinPACE | 623 (18) | 0.73 (0.41,1.32) | 575 (23) | 1.03 (0.59,1.78) | 0.782 | 617 (23) | 1.25 (0.70,2.22) | 540 (24) | 1.50 (0.85,2.65) | 0.223 |
|  | DNAmTL | 596 (28) | 1.40 (0.81,2.41) | 635 (18) | 0.83 (0.45,1.52) | 0.640 | 568 (22) | 1.14 (0.64,2.03) | 659 (24) | 1.07 (0.61,1.88) | 0.993 |
|  | PCGrimAge | 588 (13) | 0.54 (0.28,1.04) | 608 (27) | 1.11 (0.66,1.88) | 0.290 | 575 (15) | 0.70 (0.37,1.30) | 547 (25) | 1.25 (0.73,2.13) | 0.251 |
| Induced preterm birth | Hannum | 618 (17) | 3.18 (1.31,7.71) | 542 (8) | 1.68 (0.61,4.67) | 0.511 | 623 (7) | 0.48 (0.20,1.15) | 551 (7) | 0.54 (0.22,1.30) | 0.630 |
|  | Horvath | 539 (8) | 0.73 (0.29,1.83) | 888 (13) | 0.71 (0.31,1.59) | 0.837 | 505 (11) | 2.08 (0.76,5.67) | 875 (15) | 1.63 (0.63,4.23) | 0.557 |
|  | Levine | 510 (11) | 1.34 (0.60,3.02) | 638 (8) | 0.77 (0.32,1.88) | 0.429 | 471 (11) | 1.67 (0.72,3.89) | 696 (10) | 1.02 (0.43,2.41) | 0.287 |
|  | DunedinPACE | 615 (10) | 0.84 (0.38,1.88) | 559 (7) | 0.65 (0.26,1.59) | 0.172 | 604 (10) | 0.90 (0.40,2.03) | 523 (7) | 0.73 (0.29,1.80) | 0.611 |
|  | DNAmTL | 579 (11) | 2.04 (0.78,5.29) | 631 (14) | 2.39 (0.96,5.95) | 0.344 | 557 (11) | 1.48 (0.62,3.51) | 646 (11) | 1.27 (0.54,3.01) | 0.208 |
|  | PCGrimAge | 587 (12) | 1.06 (0.49,2.29) | 586 (5) | 0.44 (0.16,1.21) | 0.132 | 571 (11) | 1.49 (0.64,3.45) | 532 (10) | 1.45 (0.61,3.44) | 0.917 |
| Post-term | Hannum | 660 (59) | 1.24 (0.86,1.81) | 579 (45) | 1.07 (0.72,1.59) | 0.623 | 665 (49) | 0.86 (0.59,1.25) | 592 (48) | 0.95 (0.65,1.40) | 0.280 |
|  | Horvath | 564 (42) | 0.88 (0.57,1.35) | 951 (76) | 0.95 (0.65,1.38) | 0.791 | 525 (31) | 0.78 (0.49,1.26) | 951 (91) | 1.32 (0.91,1.91) | 0.732 |
|  | Levine | 550 (51) | 1.45 (0.97,2.15) | 689 (59) | 1.32 (0.91,1.94) | 0.638 | 494 (34) | 0.90 (0.59,1.39) | 756 (70) | 1.25 (0.87,1.78) | 0.944 |
|  | DunedinPACE | 665 (60) | 1.28 (0.88,1.87) | 599 (47) | 1.10 (0.74,1.64) | 0.270 | 645 (51) | 1.08 (0.74,1.58) | 568 (52) | 1.27 (0.86,1.86) | 0.493 |
|  | DNAmTL | 609 (41) | 0.80 (0.54,1.21) | 676 (59) | 1.07 (0.74,1.54) | 0.783 | 597 (51) | 1.14 (0.77,1.69) | 691 (56) | 1.08 (0.74,1.58) | 0.500 |
|  | PCGrimAge | 620 (45) | 0.91 (0.61,1.35) | 636 (55) | 1.10 (0.76,1.59) | 0.112 | 592 (32) | 0.65 (0.42,1.00) | 584 (62) | 1.36 (0.95,1.93) | 0.519 |
| sga | Hannum | 633 (69) | 1.38 (0.96,1.97) | 541 (46) | 1.05 (0.70,1.55) | 0.864 | 630 (60) | 1.09 (0.75,1.56) | 554 (52) | 1.07 (0.73,1.56) | 0.886 |
|  | Horvath | 536 (52) | 1.08 (0.72,1.63) | 891 (79) | 0.98 (0.67,1.43) | 0.569 | 499 (48) | 1.03 (0.68,1.55) | 902 (80) | 0.94 (0.65,1.35) | 0.502 |
|  | Levine | 512 (57) | 1.20 (0.83,1.72) | 633 (45) | 0.73 (0.50,1.07) | 0.204 | 461 (46) | 1.13 (0.76,1.67) | 711 (64) | 1.01 (0.71,1.44) | 0.786 |
|  | DunedinPACE | 635 (53) | 0.80 (0.56,1.16) | 546 (47) | 0.83 (0.57,1.22) | 0.195 | 608 (60) | 1.02 (0.72,1.45) | 521 (40) | 0.77 (0.52,1.15) | 0.716 |
|  | DNAmTL | 577 (54) | 0.99 (0.68,1.43) | 641 (55) | 0.90 (0.62,1.30) | 0.719 | 564 (48) | 0.82 (0.56,1.20) | 643 (56) | 0.84 (0.59,1.21) | 0.594 |
|  | PCGrimAge | 581 (57) | 1.17 (0.80,1.69) | 592 (55) | 1.10 (0.76,1.59) | 0.918 | 549 (47) | 0.75 (0.52,1.08) | 557 (39) | 0.60 (0.41,0.89) | 0.579 |
| lga | Hannum | 634 (70) | 0.88 (0.63,1.21) | 562 (67) | 0.96 (0.69,1.33) | 0.154 | 643 (73) | 0.91 (0.66,1.25) | 568 (66) | 0.93 (0.67,1.30) | 0.070 |
|  | Horvath | 539 (55) | 0.76 (0.53,1.11) | 924 (112) | 0.93 (0.67,1.27) | 0.045 | 509 (58) | 0.83 (0.57,1.19) | 924 (102) | 0.80 (0.58,1.09) | 0.540 |
|  | Levine | 519 (64) | 1.28 (0.90,1.81) | 682 (94) | 1.45 (1.06,1.99) | 0.037 | 473 (58) | 1.03 (0.72,1.45) | 730 (83) | 0.94 (0.69,1.29) | 0.292 |
|  | DunedinPACE | 646 (64) | 0.84 (0.60,1.18) | 582 (83) | 1.28 (0.93,1.76) | 0.090 | 620 (72) | 1.13 (0.81,1.57) | 560 (79) | 1.41 (1.02,1.95) | 0.759 |
|  | DNAmTL | 594 (71) | 0.94 (0.68,1.30) | 657 (71) | 0.84 (0.60,1.16) | 0.378 | 585 (69) | 1.08 (0.77,1.52) | 673 (86) | 1.18 (0.86,1.63) | 0.745 |
|  | PCGrimAge | 593 (69) | 1.02 (0.73,1.42) | 614 (77) | 1.11 (0.80,1.53) | 0.177 | 572 (70) | 1.00 (0.73,1.38) | 581 (63) | 0.87 (0.63,1.22) | 0.463 |
| preeclampsia | Hannum | 703 (25) | 1.60 (0.88,2.90) | 608 (24) | 1.78 (0.98,3.25) | 0.666 | 704 (23) | 0.94 (0.54,1.64) | 620 (16) | 0.74 (0.40,1.37) | 0.830 |
|  | Horvath | 591 (14) | 0.59 (0.30,1.14) | 1004 (31) | 0.77 (0.45,1.32) | 0.452 | 557 (14) | 0.75 (0.38,1.49) | 1005 (34) | 1.02 (0.58,1.77) | 0.536 |
|  | Levine | 576 (12) | 0.61 (0.31,1.21) | 727 (27) | 1.11 (0.66,1.89) | 0.538 | 520 (15) | 1.01 (0.53,1.94) | 794 (29) | 1.29 (0.75,2.23) | 0.325 |
|  | DunedinPACE | 699 (17) | 0.61 (0.34,1.10) | 630 (18) | 0.72 (0.40,1.29) | 0.820 | 681 (22) | 1.14 (0.64,2.03) | 600 (21) | 1.24 (0.69,2.22) | 0.295 |
|  | DNAmTL | 648 (23) | 1.15 (0.65,2.03) | 712 (20) | 0.90 (0.50,1.63) | 0.122 | 633 (17) | 0.89 (0.48,1.66) | 730 (27) | 1.24 (0.71,2.15) | 0.288 |
|  | PCGrimAge | 650 (24) | 1.31 (0.74,2.31) | 669 (20) | 1.05 (0.58,1.91) | 0.516 | 620 (16) | 0.66 (0.36,1.19) | 620 (16) | 0.66 (0.36,1.19) | 0.176 |

^a^ Test for non-linear relationship conducted by adding a second order term for the measures of epigenetic age acceleration in the model .

Table S11 Differences in standardized birthweight and gestational age stratified by offspring sex

| Outcome | Epigenetic age clock | Mothers | | | Fathers | | |
| --- | --- | --- | --- | --- | --- | --- | --- |
|  |  | Male offspring  (n=1,068) | Female offspring  (n=1,130) | p-value for test of non-linear relationship^a^ | Male offspring  (n=1,062) | Female offspring  (n1,131) | p-value for test of non-linear relationship^a^ |
|  |  | Mean difference  (95% CI) | Mean difference  (95% CI) |  | Mean difference  (95% CI) | Mean difference  (95% CI) |  |
| Gestational age in days | Hannum | -0.32 (-1.08,0.43) | -0.31 (-0.95,0.32) | 0.983 | -0.17 (-0.91,0.57) | -0.14 (-0.83,0.55) | 0.256 |
|  | Horvath | -1.09 (-1.81,-0.36) | 0.11 (-0.55,0.77) | 0.017 | 0.07 (-0.66,0.80) | 0.32 (-0.35,1.00) | 0.224 |
|  | Levine | -1.09 (-1.88,-0.29) | -0.57 (-1.18,0.04) | 0.313 | 0.19 (-0.53,0.92) | -0.37 (-1.02,0.28) | 0.566 |
|  | DunedinPACE | -0.45 (-1.21,0.31) | -0.98 (-1.66,-0.30) | 0.308 | 0.10 (-0.65,0.86) | -0.19 (-0.85,0.47) | 0.946 |
|  | DNAmTL | 0.51 (-0.22,1.24) | 0.75 (0.08,1.42) | 0.635 | -0.20 (-0.94,0.54) | 0.71 (0.04,1.38) | 0.075 |
|  | PCGrimAge | -0.87 (-1.59,-0.14) | -0.27 (-0.95,0.41) | 0.239 | 0.10 (-0.65,0.86) | -0.17 (-0.86,0.53) | 0.604 |
| Standardized birthweight | Hannum | 0.02 (-0.04,0.08) | 0.05 (-0.00,0.11) | 0.396 | 0.01 (-0.05,0.07) | 0.05 (-0.01,0.11) | 0.636 |
|  | Horvath | -0.01 (-0.07,0.05) | 0.03 (-0.03,0.09) | 0.362 | -0.04 (-0.10,0.01) | 0.04 (-0.02,0.09) | 0.824 |
|  | Levine | 0.04 (-0.03,0.10) | 0.03 (-0.02,0.09) | 0.889 | -0.01 (-0.07,0.04) | 0.01 (-0.05,0.06) | 0.821 |
|  | DunedinPACE | 0.05 (-0.01,0.11) | 0.11 (0.05,0.17) | 0.136 | -0.01 (-0.07,0.05) | -0.00 (-0.06,0.05) | 0.413 |
|  | DNAmTL | -0.01 (-0.07,0.04) | -0.02 (-0.08,0.04) | 0.842 | 0.02 (-0.04,0.07) | -0.04 (-0.10,0.02) | 0.203 |
|  | PCGrimAge | -0.00 (-0.06,0.06) | 0.05 (-0.01,0.11) | 0.203 | -0.03 (-0.09,0.03) | 0.03 (-0.03,0.09) | 0.153 |

^a^ Test for non-linear relationship conducted by adding a second order term for the measures of epigenetic age acceleration in the model .

Table S12 Differences in the odds of adverse birth outcomes stratified by offspring sex

| Outcome | Epigenetic age clock | Mothers | | | | | Fathers | | | | |
| --- | --- | --- | --- | --- | --- | --- | --- | --- | --- | --- | --- |
|  |  | Male offspring | | Female offspring | | p-value for test of non-linear relationship^a^ | Male offspring | | Female offspring | | p-value for test of non-linear relationship^a^ |
|  |  | N (n cases) | OR (95% CI) | N (n cases) | OR (95% CI) |  | N (n cases) | OR (95% CI) | N (n cases) | OR (95% CI) |  |
| Preterm birth | Hannum | 1,058 (59) | 0.83 (0.64,1.09) | 967 (45) | 0.99 (0.75,1.31) | 0.371 | 961 (45) | 1.10 (0.85,1.44) | 1,058 (59) | 0.87 (0.65,1.16) | 0.660 |
|  | Horvath | 1,058 (59) | 1.30 (0.99,1.71) | 967 (45) | 0.95 (0.71,1.26) | 0.116 | 961 (45) | 0.93 (0.71,1.20) | 1,058 (59) | 1.14 (0.84,1.54) | 0.798 |
|  | Levine | 1,058 (59) | 1.16 (0.87,1.56) | 967 (45) | 1.33 (0.97,1.83) | 0.542 | 961 (45) | 0.98 (0.76,1.27) | 1,058 (59) | 1.09 (0.81,1.46) | 0.315 |
|  | DunedinPACE | 1,058 (59) | 1.07 (0.81,1.40) | 967 (45) | 1.17 (0.86,1.58) | 0.658 | 961 (45) | 1.04 (0.80,1.35) | 1,058 (59) | 0.99 (0.74,1.33) | 0.430 |
|  | DNAmTL | 1,058 (59) | 0.91 (0.70,1.18) | 967 (45) | 0.84 (0.63,1.12) | 0.682 | 961 (45) | 1.13 (0.87,1.47) | 1,058 (59) | 0.96 (0.71,1.31) | 0.230 |
|  | PCGrimAge | 1,058 (59) | 1.30 (1.01,1.67) | 967 (45) | 0.96 (0.71,1.30) | 0.132 | 961 (45) | 1.14 (0.88,1.48) | 1,058 (59) | 1.10 (0.82,1.48) | 0.856 |
| Spontaneous preterm birth | Hannum | 1,044 (45) | 0.90 (0.67,1.22) | 949 (27) | 1.17 (0.79,1.74) | 0.307 | 943 (27) | 0.98 (0.74,1.32) | 1,044 (45) | 1.00 (0.70,1.44) | 0.936 |
|  | Horvath | 1,044 (45) | 1.24 (0.91,1.68) | 949 (27) | 1.02 (0.71,1.48) | 0.437 | 943 (27) | 1.15 (0.85,1.54) | 1,044 (45) | 1.10 (0.75,1.60) | 0.985 |
|  | Levine | 1,044 (45) | 1.21 (0.87,1.68) | 949 (27) | 1.91 (1.25,2.90) | 0.095 | 943 (27) | 1.08 (0.80,1.46) | 1,044 (45) | 0.94 (0.64,1.37) | 0.336 |
|  | DunedinPACE | 1,044 (45) | 1.10 (0.81,1.51) | 949 (27) | 1.37 (0.93,2.01) | 0.392 | 943 (27) | 0.96 (0.71,1.29) | 1,044 (45) | 1.22 (0.82,1.82) | 0.865 |
|  | DNAmTL | 1,044 (45) | 0.85 (0.63,1.14) | 949 (27) | 0.74 (0.51,1.06) | 0.545 | 943 (27) | 1.16 (0.86,1.55) | 1,044 (45) | 0.90 (0.62,1.33) | 0.564 |
|  | PCGrimAge | 1,044 (45) | 1.44 (1.09,1.90) | 949 (27) | 1.24 (0.86,1.78) | 0.515 | 943 (27) | 1.03 (0.77,1.38) | 1,044 (45) | 1.02 (0.70,1.49) | 0.684 |
| Induced preterm birth | Hannum | 940 (18) | 0.65 (0.38,1.09) | 1,013 (14) | 0.85 (0.63,1.16) | 0.381 | 934 (18) | 1.10 (0.65,1.86) | 1,013 (14) | 0.78 (0.48,1.28) | 0.491 |
|  | Horvath | 940 (18) | 1.54 (0.87,2.71) | 1,013 (14) | 0.85 (0.55,1.31) | 0.104 | 934 (18) | 0.84 (0.52,1.38) | 1,013 (14) | 1.03 (0.65,1.62) | 0.689 |
|  | Levine | 940 (18) | 1.02 (0.58,1.82) | 1,013 (14) | 0.90 (0.66,1.22) | 0.687 | 934 (18) | 0.93 (0.53,1.62) | 1,013 (14) | 1.11 (0.70,1.75) | 0.559 |
|  | DunedinPACE | 940 (18) | 0.95 (0.55,1.65) | 1,013 (14) | 0.92 (0.57,1.47) | 0.916 | 934 (18) | 1.08 (0.64,1.83) | 1,013 (14) | 0.94 (0.60,1.48) | 0.359 |
|  | DNAmTL | 940 (18) | 1.13 (0.66,1.93) | 1,013 (14) | 1.02 (0.65,1.61) | 0.780 | 934 (18) | 0.97 (0.58,1.62) | 1,013 (14) | 1.24 (0.77,1.98) | 0.235 |
|  | PCGrimAge | 940 (18) | 0.89 (0.52,1.52) | 1,013 (14) | 0.62 (0.37,1.03) | 0.343 | 934 (18) | 1.00 (0.58,1.70) | 1,013 (14) | 1.06 (0.68,1.66) | 0.639 |
| Post-term | Hannum | 1,020 (98) | 0.87 (0.68,1.12) | 1,067 (68) | 0.95 (0.79,1.15) | 0.581 | 1,014 (98) | 1.37 (1.08,1.72) | 1,068 (69) | 1.25 (1.02,1.53) | 0.354 |
|  | Horvath | 1,020 (98) | 1.03 (0.81,1.32) | 1,067 (68) | 1.05 (0.86,1.29) | 0.899 | 1,014 (98) | 1.20 (0.95,1.52) | 1,068 (69) | 1.01 (0.82,1.25) | 0.905 |
|  | Levine | 1,020 (98) | 0.84 (0.65,1.09) | 1,067 (68) | 0.97 (0.81,1.16) | 0.379 | 1,014 (98) | 1.23 (0.95,1.60) | 1,068 (69) | 1.17 (0.94,1.45) | 0.749 |
|  | DunedinPACE | 1,020 (98) | 0.96 (0.75,1.24) | 1,067 (68) | 0.89 (0.72,1.09) | 0.627 | 1,014 (98) | 1.27 (1.00,1.60) | 1,068 (69) | 1.15 (0.94,1.41) | 0.291 |
|  | DNAmTL | 1,020 (98) | 1.10 (0.86,1.41) | 1,067 (68) | 1.07 (0.87,1.32) | 0.880 | 1,014 (98) | 0.98 (0.77,1.25) | 1,068 (69) | 1.14 (0.93,1.41) | 0.730 |
|  | PCGrimAge | 1,020 (98) | 0.93 (0.73,1.20) | 1,067 (68) | 1.13 (0.92,1.39) | 0.236 | 1,014 (98) | 1.20 (0.94,1.53) | 1,068 (69) | 1.17 (0.95,1.45) | 0.560 |
| sga | Hannum | 950 (88) | 0.94 (0.75,1.17) | 1,008 (91) | 0.79 (0.63,0.99) | 0.294 | 945 (89) | 0.94 (0.76,1.17) | 1,009 (91) | 1.07 (0.86,1.33) | 0.990 |
|  | Horvath | 950 (88) | 0.87 (0.70,1.07) | 1,008 (91) | 1.00 (0.80,1.24) | 0.362 | 945 (89) | 1.01 (0.81,1.24) | 1,009 (91) | 0.91 (0.73,1.13) | 0.091 |
|  | Levine | 950 (88) | 0.78 (0.62,0.98) | 1,008 (91) | 0.98 (0.78,1.23) | 0.165 | 945 (89) | 1.00 (0.81,1.24) | 1,009 (91) | 1.00 (0.80,1.24) | 0.479 |
|  | DunedinPACE | 950 (88) | 1.09 (0.87,1.37) | 1,008 (91) | 1.04 (0.84,1.30) | 0.785 | 945 (89) | 1.04 (0.84,1.29) | 1,009 (91) | 0.79 (0.62,1.00) | 0.632 |
|  | DNAmTL | 950 (88) | 1.06 (0.86,1.32) | 1,008 (91) | 1.01 (0.81,1.26) | 0.758 | 945 (89) | 0.95 (0.76,1.18) | 1,009 (91) | 0.88 (0.70,1.10) | 0.428 |
|  | PCGrimAge | 950 (88) | 0.96 (0.78,1.19) | 1,008 (91) | 1.06 (0.85,1.31) | 0.532 | 945 (89) | 0.90 (0.73,1.11) | 1,009 (91) | 1.18 (0.94,1.47) | 0.088 |
| lga | Hannum | 979 (117) | 0.95 (0.79,1.16) | 1,039 (122) | 1.07 (0.89,1.30) | 0.389 | 972 (116) | 1.08 (0.89,1.31) | 1,040 (122) | 1.08 (0.89,1.30) | 0.679 |
|  | Horvath | 979 (117) | 0.99 (0.82,1.19) | 1,039 (122) | 1.13 (0.93,1.36) | 0.342 | 972 (116) | 0.97 (0.81,1.16) | 1,040 (122) | 1.02 (0.84,1.24) | 0.709 |
|  | Levine | 979 (117) | 1.00 (0.81,1.22) | 1,039 (122) | 1.09 (0.90,1.32) | 0.532 | 972 (116) | 0.92 (0.77,1.11) | 1,040 (122) | 0.97 (0.81,1.17) | 0.639 |
|  | DunedinPACE | 979 (117) | 1.03 (0.85,1.25) | 1,039 (122) | 1.41 (1.16,1.71) | 0.027 | 972 (116) | 0.93 (0.77,1.12) | 1,040 (122) | 0.99 (0.82,1.19) | 0.782 |
|  | DNAmTL | 979 (117) | 0.95 (0.78,1.14) | 1,039 (122) | 1.07 (0.88,1.29) | 0.385 | 972 (116) | 0.88 (0.73,1.07) | 1,040 (122) | 0.98 (0.81,1.18) | 0.976 |
|  | PCGrimAge | 979 (117) | 0.97 (0.81,1.18) | 1,039 (122) | 1.18 (0.97,1.42) | 0.167 | 972 (116) | 1.06 (0.88,1.28) | 1,040 (122) | 1.02 (0.84,1.24) | 0.685 |
| preeclampsia | Hannum | 1,062 (37) | 1.37 (0.94,1.98) | 1,131 (32) | 0.95 (0.71,1.26) | 0.127 | 1,062 (37) | 1.07 (0.75,1.52) | 1,131 (32) | 0.95 (0.68,1.33) | 0.530 |
|  | Horvath | 1,062 (37) | 1.26 (0.88,1.80) | 1,131 (32) | 1.02 (0.74,1.40) | 0.393 | 1,062 (37) | 0.99 (0.70,1.39) | 1,131 (32) | 1.16 (0.83,1.62) | 0.506 |
|  | Levine | 1,062 (37) | 1.25 (0.85,1.85) | 1,131 (32) | 1.11 (0.80,1.54) | 0.647 | 1,062 (37) | 1.10 (0.78,1.55) | 1,131 (32) | 1.00 (0.72,1.39) | 0.276 |
|  | DunedinPACE | 1,062 (37) | 0.94 (0.65,1.35) | 1,131 (32) | 1.34 (0.96,1.86) | 0.154 | 1,062 (37) | 1.17 (0.81,1.67) | 1,131 (32) | 1.00 (0.73,1.39) | 0.706 |
|  | DNAmTL | 1,062 (37) | 0.97 (0.68,1.37) | 1,131 (32) | 0.93 (0.68,1.29) | 0.873 | 1,062 (37) | 1.03 (0.73,1.46) | 1,131 (32) | 0.93 (0.67,1.29) | 0.545 |
|  | PCGrimAge | 1,062 (37) | 0.87 (0.61,1.24) | 1,131 (32) | 1.03 (0.74,1.43) | 0.483 | 1,062 (37) | 1.38 (0.94,2.01) | 1,131 (32) | 1.04 (0.75,1.45) | 0.622 |

^a^ Test for non-linear relationship conducted by adding a second order term for the measures of epigenetic age acceleration in the model .
